# Ligand-Targeted Delivery of Photosensitizers for Cancer Treatment

**DOI:** 10.3390/molecules25225317

**Published:** 2020-11-14

**Authors:** Piotr Gierlich, Ana I. Mata, Claire Donohoe, Rui M. M. Brito, Mathias O. Senge, Lígia C. Gomes-da-Silva

**Affiliations:** 1 CQC, Coimbra Chemistry Center, Department of Chemistry, University of Coimbra, 3000-435 Coimbra, Portugal; gierlicp@tcd.ie (P.G.); ana.mata@student.uc.pt (A.I.M.); donohoc2@tcd.ie (C.D.); brito@ci.uc.pt (R.M.M.B.); 2 Medicinal Chemistry, Trinity Translational Medicine Institute, Trinity Centre for Health Sciences, Trinity College Dublin, The University of Dublin, St. James’s Hospital, D08W9RT Dublin, Ireland; Mathias.Senge@tcd.ie; 3 BSIM Therapeutics, Instituto Pedro Nunes, 3030-199 Coimbra, Portugal

**Keywords:** photodynamic therapy, cancer, drug delivery, active targeting, nanocarriers

## Abstract

Photodynamic therapy (PDT) is a promising cancer treatment which involves a photosensitizer (PS), light at a specific wavelength for PS activation and oxygen, which combine to elicit cell death. While the illumination required to activate a PS imparts a certain amount of selectivity to PDT treatments, poor tumor accumulation and cell internalization are still inherent properties of most intravenously administered PSs. As a result, common consequences of PDT include skin photosensitivity. To overcome the mentioned issues, PSs may be tailored to specifically target overexpressed biomarkers of tumors. This active targeting can be achieved by direct conjugation of the PS to a ligand with enhanced affinity for a target overexpressed on cancer cells and/or other cells of the tumor microenvironment. Alternatively, PSs may be incorporated into ligand-targeted nanocarriers, which may also encompass multi-functionalities, including diagnosis and therapy. In this review, we highlight the major advances in active targeting of PSs, either by means of ligand-derived bioconjugates or by exploiting ligand-targeting nanocarriers.

## 1. Introduction

A critical limiting factor of cancer treatment’s success is the lack of specificity associated with many traditional cancer therapeutics. Moreover, most anti-cancer drugs accumulate in normal and cancer tissues indiscriminately. Damage is instigated in proportion to the sensitivity of the tissue exposed [1]. This not only leads to significant, often debilitating side effects, but also to a decreased therapeutic efficacy [2]. Due to these obstacles, intense research is focused on the development of strategies to deliver effective therapeutic concentrations of anti-cancer agents specifically to the tumor, thereby increasing their therapeutic efficacy while reducing toxicity [3,4].

Targeted drug delivery in the context of cancer is mainly achieved by two approaches: passive and active targeting. The first is highly dependent on the physicochemical properties of drugs/nanocarriers and the pathophysiological features of the tumors [5]. It is proposed that the leaky and discontinuous tumor endothelium, in conjunction with poor tumor lymphatic drainage, naturally favors the accumulation of drugs/nanocarriers in tumors, a phenomenon known as the enhanced permeability and retention (EPR) effect [6]. In contrast, active targeting refers to the specific interactions, at a molecular level, between a drug or its delivery system and the target cells (e.g., cancer cells), usually due to specific ligand–receptor interactions [7]. Extensive genome sequencing and proteomic exploration have caused a large number of biomarkers overexpressed in cancer cells to be discovered as suitable receptors for active targeting [8]. Active targeting intends not only to enhance tumor accumulation but also to increase intracellular delivery of the drugs through exploitation of receptor mediated endocytosis [9]. Although major improvements for the internalization of drugs or their delivery systems (e.g., nanocarriers) by cancer cells have been shown in vitro by means of different targeting moieties, limited success has been observed with in vivo cancer mouse models. Some studies have shown that upon systemic administration, targeted and non-targeted drugs/nanocarriers unexpectedly exhibited similar tumor accumulation. This clearly indicates that tumor accumulation of both targeted and non-targeted drugs/nanocarriers is highly dependent on the tumor pathophysiology, and therefore, on the EPR effect, rather than solely the presence of a ligand targeting the cancer cells [10,11]. Even targeted radiotherapy which utilizes high binding affinity antibody ligands only achieves below 0.01% of dose administered localized in the tumor [12,13]. Nevertheless, at the tumor level, the presence of the targeting ligand is crucial to enhance drug/nanocarrier internalization by the cancer cells. This is expected to correlate with improved therapeutic outcomes when compared with the non-targeted controls [10,11]. In addition, other cell populations of the tumor microenvironment (TME) rather than cancer cells have received great attention. For instance, targeting tumor endothelial cells is becoming popular, as they are considered important for angiogenesis, subsequent tumor growth and metastasis formation. Additionally, endothelial cells are readily accessible to any drug/nanocarriers injected in the vascular compartment, while cancer cell targeting is dependent on the drug extravasation from tumor vasculature [14].

Photodynamic therapy (PDT) is a promising and non-invasive anti-cancer treatment that may be potentiated by ligand-targeted strategies [15]. It relies on the interaction between light, a photosensitizer (PS) pro-drug and ground state molecular oxygen, which combine to provide a therapeutic effect mediated by singlet oxygen and/or other reactive oxygen species (ROS) [16,17]. Photodynamic action may proceed via two known principal paths of reaction, both very dependent on the oxygen content present in cells [18]. Absorption of a photon by the PS at a specific wavelength causes activation from the ground state to a short-lived excited state. The excited PS may decay—emitting fluorescence—and return to the ground state, or it can undergo intersystem crossing to form a relatively long-lived triplet state. The triplet state may also decay radiatively, emitting phosphorescence. This is a spin forbidden process, and thus, occurs slowly. Crucially, however, it may interact with molecular oxygen in a type I reaction to transfer an electron to surrounding biomolecules to produce ROS [15,19]. Alternatively, in a type II reaction, energy of the excited PS may be directly transferred to ground-state molecular oxygen, producing singlet oxygen, ^1^O_2_ [15,16,18,19].

An ideal PS for the treatment of solid tumors should absorb light, with a high molar absorptivity, at a wavelength between 650 and 850 nm. high light penetration through human tissues is achieved while activation of biomolecules, for example, hemoglobin, is avoided. At the clinical level, most PSs are administered systemically. Following a certain interval of time, the drug to light interval (DLI), the tumor is illuminated, causing the photo-activation of the PS accumulated in the tumor microenvironment [19]. Tumor destruction is attained via a number of downstream targets, including cancer cells, tumor vasculature and the immune host system [20]. The primary site of damage is generally considered to coincide with the site of PS accumulation due to the short lifetime of ROS [21,22]. The accumulation of the PS in the tumor is highly dependent on the DLI. Prolonged DLI aims for the optimal distribution of the compound in cellular compartments (cellular-PDT), while the tumor vasculature tends to be targeted by PDT using shorter DLI (vascular PDT) [19]. In addition to irreversible damage to the cancer cells and tumor microvasculature, PDT may also activate the immune system against tumor antigens, which can lead to the induction of anti-tumor immunity [22,23].

Despite extensive research, PDT has yet to gain clinical acceptance as a first line anti-cancer therapy [20]. Porfimer sodium (Photofrin, “haematoporphyrin derivative”), temoporfin (Foscan, 5,10,15,20-tetrakis(3-hydroxyphenyl)chlorin), 5-aminolevulinic acid (5-ALA) and talaporfin (Laserphyrin, “mono-L-aspartyl chlorin e_6_”) are the PSs already in clinical practice for cancer treatment [24]. Formulation problems and difficulties in planning and monitoring the clinical administration have been significant challenges [19,25].

**Figure 1:**
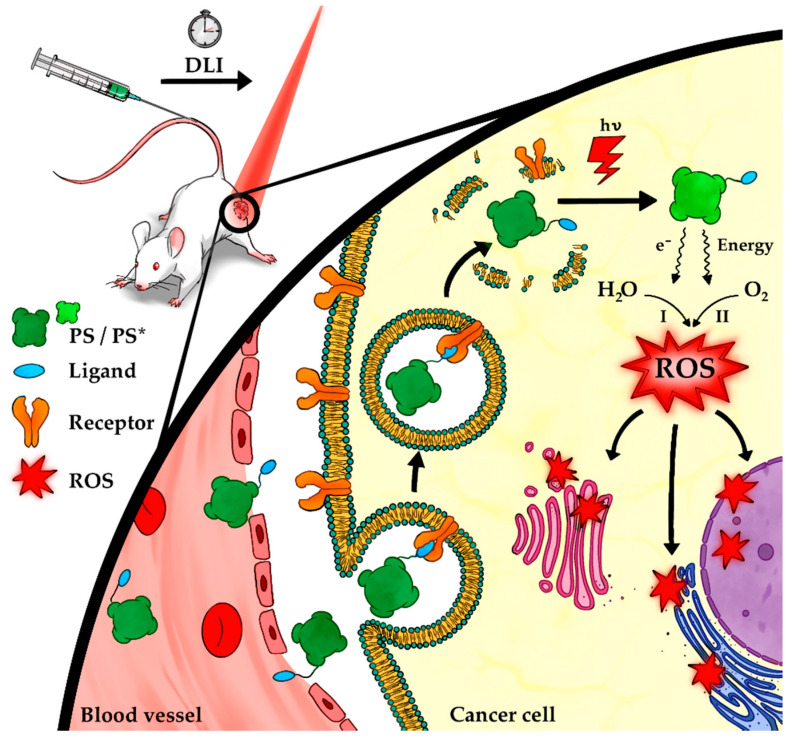
Ligand-targeted strategies may ensure effective delivery of IV-administered photosensitizers (PSs) to cells of the tumor microenvironment. Upon intravenous administration, a ligand-targeted PS is expected to be in circulation for adequate time to allow extravasation through endothelial fenestrations of the angiogenic tumor blood vessels into the tumor mass. Upon tumor accumulation, the targeting moiety attached to the PS is recognized by receptors overexpressed on the surfaces of cancer or other stromal cells, leading to endocytosis-mediated internalization of the PS. When the targeting-ligand and/or drug delivery carrier exhibits fusogenic properties that can destabilize the endocytic vesicles, the PS is released into the cell cytosol with further accumulation in different organelles. However, the PS may remain entrapped at the endocytic compartment until the illumination time. After a certain time (drug-to-light interval, DLI) which typically corresponds to the time that allows the highest tumor accumulation, illumination of tumors is performed with a laser at an appropriate wavelength. Photons are then absorbed by the PS which interact with molecular oxygen in type I and/or II reactions. Local generation of singlet oxygen, ^1^O_2_, and/or different reactive oxygen species (ROS) oxidizes biomolecules in their vicinity. Finally, the generated oxidative stress and associated damage culminate in cancer cell death via different mechanisms.

PDT treatments already offer some tumor selectivity and specificity as a function of the illumination region. Additionally, PSs (namely amphiphilic and lipophilic PSs) are known to have preferential accumulation at tumor sites due to their interaction with low density lipoproteins (LDL), which have overexpressed receptors on cancer cells [26]. Despite this, significant improvements for PDT treatment might be achieved by means of ligand-targeted strategies. Higher and more specific PS tumor accumulation is expected to enhance tumor destruction while avoiding skin photosensitivity [19,25,27] (Figure 1).

In this review, the major advances regarding active targeting delivery of PSs, either by means of ligand-derived PS bioconjugates or by taking advantage of ligand-targeting nanocarriers, will be discussed in the context of cellular-PDT for cancer treatment.

## 2. Targeting Approaches in the Context of Cancer

### 2.1. Targeting Different Populations of the Tumor Microenvironment

A tumor is not only a group of cancer cells proliferating in an uncontrolled manner but rather a complex tissue composed of different types of cells. These cells include cancer cells, cancer stem cells, endothelial cells, pericytes, cancer-associated fibroblasts and different types of immune infiltrating cells [28]. Collaborative interactions between cancer cells, associated stroma cells and the extracellular matrix form the tumor microenvironment, which governs disease initiation, progression and metastasis formation [29]. An awareness of the complexity of the tumor microenvironment is gaining acceptance as a necessary consideration for the design of novel cancer therapies. Indeed, a successful therapeutic approach should take into consideration the tumor microenvironment dynamics, and potentially, strategies that target different tumor cell populations may enhance therapeutic outcomes [29].

#### 2.1.1. Targeting Cancer Cells and Cancer Stem Cells (CSCs)

The majority of the targeting approaches investigated to date are aimed at targeting cancer cells. Cancer cells express a large number of cell-surface receptors, often overexpressed, to fulfil the needs of tumor growth, migration, invasion and metastasis. Hence, these receptors can serve as suitable candidates for ligand-targeted cancer therapy.

Growth factor receptors, such as folate (FA) and transferrin (Tf) receptors, are regularly probed cancer cell targets owing to their overexpression in cancers of different histological origin. Different isoforms of the FA receptor (FR) exist with the α form present in several types of cancer cells, while the β form is mainly found on tumor-associated macrophages and monocytes [30]. FRα confers advantages for tumor growth, even in microenvironments with limited folate availability [31]. Similarly, the human Tf receptor 1 (TfR1) is an example of a transmembrane glycoprotein receptor often overexpressed on the surfaces of cancer cells [32], which ensures iron uptake by cancer cells, thereby playing a crucial role in cell growth [33]. FA and Tf targeting have proved to significantly enhance internalization in cancer cells. However, a certain degree of non-specificity may arise, as expression of those receptors in healthy tissues also occurs. Moreover, folate from diet can also be found in significant levels in body fluids, which will compete with the targeting therapy [34].

Other approaches based on the recent advances in antibody engineering and phage-display technology have been used to achieve targeting strategies with higher selectivity and specificity. For instance, monoclonal antibodies (mAbs), antibody fragments and nanobodies have been used to target cancer cell receptors with higher specificity. In this regard, the epidermal growth factor receptor (EGFR) is one of the most studied. EGFR is a receptor tyrosine kinase (RTK) expressed on normal human cells; however, significantly higher levels of expression are correlated with malignancy in a variety of epithelial cancers [35,36]. Its activation stimulates key processes for tumor growth, such as proliferation, angiogenesis, invasion and metastasis formation [36]. Targeting of EGFR has been achieved successfully with the mAb cetuximab [35] and by means of different peptides, such as the GE11 peptide [37,38,39,40]. Human epidermal receptor-2 (HER-2), another RTK, also represents a relevant therapeutic target as it is the most common overexpressed receptor in breast cancers, while it is minimally expressed in normal tissues [35,40]. Targeted therapy using clinically approved, anti-HER-2 mAb trastuzumab is widely used for the treatment of HER-2 breast cancer [41].

Within tumors, CSCs are a small subpopulation of cells which are capable of self-renewal and differentiation into multiple cell types. CSC are highly tumorigenic and are also referred to as tumor-initiating cells. For instance, as few as 100 CSCs (isolated and identified as CD44^+^ CD24^-^) were able to induce tumor growth in non-obese diabetic/severe combined immuno-deficient (NOD/SCID) mice [42], while in a humanized mouse model (NSG^TM^), injection of 1000 melanoma CD71^+^ cells resulted in successful tumor induction [43]. The CSC tumor subpopulation is very challenging to eliminate, as they are often resistant to therapies, including chemotherapy and radiotherapy [44]. Despite this, CSC targeting presents an opportunity to fight cancer at the root by avoiding tumor relapse and metastasis formation [45].

The most common CSCs surface markers include CD44, CD133, ALDH1A1, CD34, CD24 and epithelial cell adhesion molecule (EpCAM). Among them, CD44 and CD133 are found overexpressed in different types of cancer. They are transmembrane glycoproteins with different functions that promote tumorigenesis. Targeting of CD44 may be achieved through the use of its endogenous ligand, hyaluronic acid (HA) [46], and by means of antibodies [46,47]. Other prominent markers of CSCs may be also targeted by monoclonal antibodies [48].

#### 2.1.2. Targeting Endothelial Cells from Tumor Angiogenic Blood Vessels

The tumor microenvironment offers alternative targets for tumor delivery, including endothelial cells of the tumor blood and lymphatic vessels [49]. Cancer growth relies on an ability to induce the formation of new capillaries from pre-existing vessels, a process termed angiogenesis [12]. Thus, vascular endothelial cells are an important target for cancer treatment, as impeding angiogenesis is expected to cause tumor cell death due to reduced oxygen and nutrient supply [36]. Inhibition of angiogenesis may itself be a selective process. It occurs with a limited number of physiological processes, including wound healing, ovulation and pregnancy [50]. From a practical perspective, vascular endothelial cells are more directly accessible following systemic administration, and therefore, long circulation half-lives of the targeting therapeutics might not be necessary. Slower mutation rate and reduced risk of acquired drug resistance are additional advantages of targeting endothelial cells [14,51].

The unique features of the tumor vasculature have allowed the identification of several molecular targets that can be exploited to deliver therapeutics to the vasculature. The vascular endothelial growth factor receptors (VEGFR) are a family of glycoprotein receptors with vital functions for tumor vessel angiogenesis and neovascularization. VEGF overexpression has been associated with advanced tumor progression. Thus, the VEGF/VEGFR signaling blockade is of great interest as a targeted therapy. This may be achieved through mAbs (e.g., ramacirumab and tanibirumab) for colorectal, breast and lung cancers [52].

Integrins serve as another potential target of the tumor endothelial cells. Integrins are a family of cell surface transmembrane receptors that mediate interactions between the cell cytoskeleton and the extracellular microenvironment [53]. For instance, the α_v_β_3_ integrin is highly expressed on neovascular endothelial cells and tumor cells, while lower expression is found in resting endothelial cells and most healthy tissues [36]. With regard to targeting α_v_β_3_ integrins, cyclic or linear derivatives of RGD (Arg–Gly–Asp) oligopeptides have been the most studied ligands [36]. Other ligands have included mAbs such as abituzumab, an anti-α_v_ class integrin inhibitor [54]. Abituzumab has presented a typical profile of integrin-targeting therapeutic development with promising preclinical in vivo results that demonstrated tumor growth blockade. However, late phase clinical trial outcomes were very disappointing [53,55]. In a phase 1/2 trial, combination of abituzumab with standard therapy of cetuximab plus irinotecan for the treatment of wild-type metastatic colorectal cancer compared to standard therapy alone demonstrated a lack of improvement [56]. The lack of success of integrin targeted therapies may be attributed to the potential drawback of animal models as misleading guides [57].

Vascular cell adhesion molecule-1 (VCAM-1) is a transmembrane immunoglobulin found expressed on the surfaces of tumor endothelial cells [58]. It is an optimal target due to its virtual absence from normal human vasculature [59]. VCAM-1 expression is induced by several inflammatory cytokines and plays a significant role in leukocyte recruitment to sites of tissue inflammation [60]. Additionally, during tumor migration and angiogenesis, integrins on the surfaces of tumor endothelial cells bind to VCAM-1 [59]. This binding promotes cell-to-cell adhesion and potentially extravasation of cancer cells, and therefore, metastasis formation. VCAM-1 is aberrantly expressed in breast, gastric, renal, melanoma, ovarian and colorectal cancers [58,61]. VCAM-1 may be targeted in drug delivery through the use of anti-VCAM-1 mAbs, which have been shown to enhance vascular tumor accumulation [14].

Finally, matrix metalloproteinases (MMP) are also promising targets of the tumor microenvironment. MMPs are a family of zinc-dependent endopeptidases mainly responsible for the turnover and degradation of the extracellular matrix [59]. MMPs are present in nearly all human cancer cells and their expression is correlated with metastatic potential and patient prognosis [62,63]. As an example, the MMP aminopeptidase N (APN), also known as CD13, is overexpressed on the endothelial cell surfaces of almost all major tumor forms. It has important roles in angiogenesis and tumor cell invasion. It is the receptor of the NGR peptide (Asp-Gly-Arg) and of its cyclic form. Both are widely used to target drugs towards tumor vessels [62,64].

#### 2.1.3. Simultaneous Targeting of Different Cell Populations of the Tumor Microenvironment

The targeting of a marker present on multiple tumor cell types may offer the possibility of simultaneously targeting different cells that contribute to tumor progression (Table 1).

Significant progress was achieved with the discovery of the Lyp-1 peptide by Laakkonen et al. [65], which specifically binds to p32 receptors. These are overexpressed on tumor cells, tumor-associated macrophages and tumor associated lymphatics [66]. Targeting of tumor lymphatics vessels, in addition to cancer cells, could improve significantly therapeutic effects. This is due to metastasis formation often occurring upon cancer cells’ migration through the tumor lymphatic vessels [67,68]. Lyp-1 is a cyclic, 9-amino acid peptide that co-localizes with three different lymphatic endothelial cell markers, including lymphatic vessel endothelial hyaluronic acid receptor-1 (LYVE-1), podoplanin and VEGFR3. Importantly, it does not colocalize with lymphatic vessels of normal tissue. In addition to its targeting abilities, Lyp-1 has been demonstrated to have intrinsic therapeutic activity with inhibition of breast tumor growth in vivo [69]. Based on the success obtained with Lyp-1, the p32 receptor continues to be used as part of the screening process for ligands capable of lymphatic targeting [70].

Nucleolin is an intracellular protein overexpressed on both tumor endothelial cells and cancer cells of different histological origin [71]. Recently, it was also shown as a marker of breast CSCs [72]. Thus, nucleolin targeting enables simultaneous tackling of different tumor cell populations, which is expected to bring important therapeutic benefits. Major achievements have been revealed with the F3 peptide, a synthetic 31 amino acid peptide, which is a specific ligand for nucleolin [72,73,74,75,76].

In addition to enhanced cellular internalization, both the F3 and the Lyp-1 peptides can act as tumor-penetrating peptides due to the presence of “C-end rule” (CendR) motifs within their sequences [77]. Peptides with one arginine (R) (or rarely a lysine (K)) in the C terminus with the sequence R/K/XXR/K can be recognized and internalized by neuropilin-1, which is overexpressed on tumor endothelial and cancer cells. This activates a trans-tissue transport pathway which is mediated by endocytosis and exocytosis of the targeting therapeutics through endothelial and cancer cells, thereby allowing vascular extravasation and penetration across the tumor mass. F3 and Lyp-1 peptides are expected to be cleaved by endogenous proteases, exposing their internal CendR at the C-terminus, which improves tumor penetration [78]. Other examples of peptides with multi-targeting abilities are presented in Table 1.

**Table 1:** Examples of peptides for simultaneous targeting.

Peptide	Receptor	Target Cells of TME	Ref.
Lyp-1	p32, NRP	Cancer cells, tumor lymphatic endothelial cells and tumor associated macrophages	[65,79]
F3	Nucleolin	Cancer cells, CSCs and tumor endothelial cells	[71,72,73,80,81]
iRGD	α_v_β_3_, α_v_β_5_ NRP	Cancer cells and tumor endothelial cells	[77,82]
T1	p32, NRP	Cancer cells, tumor lymphatic endothelial cells and tumor associated macrophages	[70,83]
F56	VEGFR1	Cancer cells, tumor endothelial cells, fibroblasts and tumor associated macrophages	[84]

### 2.2. Ligands for Active Targeting

Specific delivery of anti-cancer drugs to solid tumors, at relevant therapeutic doses, is still an unmet goal. One promising strategy to overcome this problem relies on the use of targeting ligands that are specifically recognized and internalized by cancer cells and/or other cells of the tumor microenvironment while avoiding healthy cells. Ligands of diverse nature (proteins, peptides, antibodies, nanobodies, etc.) have been used. Advantages and disadvantages of different classes of ligands are presented in Table 2.

### 2.3. Strategies to Identify New Ligands

The identification of new targeting moieties with higher specificity for tumors is still required for the development of targeting strategies with minimal normal tissue interaction. In the context of PDT, improved specificity is expected to significantly reduce skin sensitivity. This sensitivity is still one of the most limiting PDT side effects. Additionally, new ligands with multi-targeting abilities are also highly desirable in order to tackle the complexity and aggressiveness of the tumor microenvironment.

Phage display technology is an effective means of identifying new antibodies and peptides that can target a certain receptor. It was developed in 1985 and regained popularity with the award of half of the 2018 Nobel Prize in Chemistry to Smith and Sir Gregory Winter [116,117]. This method takes advantage of bacteriophage (viruses that infect bacteria) machinery to synthesize and display different sequences of foreign peptides or antibodies at their surfaces. Phage display screenings can be performed in situ (e.g., with a recombinant form of the target receptor), in vitro using whole cells and even in vivo. In situ phage display studies include what was reported by Li et al. over a decade ago and allowed the discovery of the GE11 peptide (YHWYGYTPQNVI) [39], still largely used to target EGFR [118,119,120]. The EGFR mimotopes, P26 (VPGWSQAFMALA) and P19 (DTDWVRMRDSAR), were also recently identified [121]. Other examples include the LS-7 (LQNAPRS) peptide which targets the CSC-associated marker CD133 [122] and the new nanobody VUN100 for targeting the G-protein coupled receptor homolog US28 that is found to be overexpressed in glioblastoma [123]. Antibodies with inhibitory activity towards integrin α11/β1 were also identified in situ [124]. Recently, in vitro phage display using gastric cancer cells allowed the identification of DE532 (VETSQYFRGTLS) and GP-5 (IHKDKNAPSLVP) peptides, and specific antibodies to target gastric cancer [125]. The RKOpep (CPKSNNGVC) peptide was selected for colorectal cancer targeting [126]. In vivo phage display was first described by Ruoslahti and co-workers in 1996 principally to identify new peptides to target brain blood vessels [127]. Later, the same group discovered the tumor-homing peptides F3 (KDEPQRRSARLSAKPAPPKPEPKPKKAPAKK) [73], LyP-1 (cCGNKRTRC) [65] and TT1 (AKRGARSTA) [83], which have multi-targeting abilities. Lately, the CSP-GD (GDALFSVPLEVY) and CSP-KQ (KQNLAEG) peptides have also been recognized by in vivo phage display as potential ligands to target human cervical cancer [128].

Additionally, computer-aided drug discovery (CADD) methods have become important tools, as they permit the simulation and/or prediction of drug-target binding, by structure or ligand-based strategies [129]. When the structure of a certain cancer target/receptor is readily available, structure-based techniques can predict possible interactions between the target and different known ligands using data-mining. On the other hand, if there is no available information on the structure of the cancer target/receptor, new ligands can be designed using available ligands as references for that target.

The work of Hidayat et al. is an example of structure-based identification of new ligands [130]. With the aim of targeting the integrin α_v_β_3_ receptor, complexes of integrin α_v_β_3_ receptor-peptidomimetic (RGD) were used to reveal the structure of the integrin binding site. Three pharmacophores were identified, which further guided the design of a new ligand. Molecular docking confirmed the interaction of the new ligand with the integrin α_v_β_3_ receptor, and molecular dynamics studies predicted a good stability of the new ligand when bound to the integrin α_v_β_3_ receptor and a good inhibitory activity. However, this ligand was never validated with in vitro or in vivo studies.

## 3. Ligand-Targeted Photosensitizers

The covalent binding of PSs to ligands, specifically recognized and internalized by cancer cells and/or other cells from the tumor microenvironment, is an approach that has been explored to enhance the selectivity and efficacy of PDT [131]. The following section highlights some of the most promising targeted PS bioconjugates.

### 3.1. Folate and Transferrin-Targeted PS

FA and Tf are among the most often used targeting ligands, included in PDT. Although more frequently explored in nanocarriers for the targeted delivery of PSs, a few works have reported the synthesis of bioconjugates with improved selectivity for cancer cells [132]. Condensation of a carboxyl group with an amino group was used by Stallivieri et al. [133] and Suvorov et al. [134] for the conjugation of different PSs (e.g., 5,10,15,20-tetraphenylporphyrin (TPP), protoporphyrin IX (Pp IX), 5,10,15,20-tetraphenylchlorin (TPC), chlorin e_6_, pheophorbide-a and zinc(II) phthalocyanines) to FA. Although lacking studies which demonstrate enhanced internalization by cancer cells, this work can serve as guideline for the synthesis of novel FA-targeted PS conjugates. Yang et al. [108] reported the conjugation of FA to a platinum porphyrin complex through an ethylenediamine linker. The activation of carboxylic acids from both FA and the platinum porphyrin complex allowed the formation of amide bonds with the linker (first the FA, followed by the PS), yielding a new FA-targeted PS selective for FRα-positive cell lines (HeLa cells). Confocal microscopy studies confirmed the endocytosis of the targeted-PS by HeLa cells, as opposed to the FRα-negative cell line (A549 cells). Phototoxicity assays showed further evidence of the PS’s selectivity, with a decrease of 78% of the viability of the FRα-positive cell line when compared to 25% of the FRα-negative line. Similarly, FA-targeted π-extended diketopyrrolopyrrole-porphyrin was also shown to be selective for FRα-positive HeLa cells [135]. The work of Liu et al. provides an important validation of the in vivo benefits of FA-targeting [136]. In a mouse model of nasopharyngeal epidermoid carcinoma, the conjugation of pyropheophorbide *a* with FA, using a 1 kDa polyethylene glycol (PEG) spacer, showed superior tumor accumulation and PDT efficacy when compared with the free or the non-targeted controls. Improvements were also noted when directly compared with the targeted-PS without the spacer PEG, highlighting the importance of the long blood circulation times needed to take advantage of the EPR effect. The PEGylated FA-targeted PS was able to eradicate subcutaneous KB tumors in BALB/c nude mice, at a considerably reduced dose (i.e., 60 nmol/mouse, DLI = 4 h, DL = 180 J/cm^2^ at 670 nm). No recurrence occurred in the 90 days following treatment, unlike the non-targeted PS and the non-PEGylated targeted PS cases [136].

In 1994, Hamblin and Newman [137] were the first to report the conjugation of Tf to a PS, namely, hematoporphyrin. Their studies showed improved internalization of Tf-targeted hematoporphyrin by cancer cells (HT29 cells) and normal fibroblast (3T3), which increased the phototoxicity of hematoporphyrin. However, the uptake was only improved in an iron-deficient environment (which upregulates Tf receptors) and in medium supplemented with polycations (to increase binding to cell membranes). With this knowledge, it was anticipated that the in vivo translation of this targeting approach would be challenged by competition with the native form of Tf. Later, Cavanaugh [86] renewed attention on TfR1 as a PDT target and developed a method for the conjugation of chlorin e_6_ to Tf, which involved the preliminary binding of the protein to quaternary amino ethyl-sephadex. After saturating the sephadex with Tf, the solution of chlorin e_6_ with its activated carboxylic acid, was added. The Tf-targeted chlorin e_6_ had the ability to kill in vitro breast cancer cells at concentrations 10–40-fold lower than the ones used with the free chlorin e_6_. More recently, Kaspler et al. [138] reported the conjugation of a ruthenium (II)-based photosensitizer (Ru(II)(4,4′-dimethyl-2,2′-bipyridine(dmb))_2_(2-(2′,2′′:5′′,2′′′-terthiophene)-imidazo[4,5-f]-[1,10]phenanthroline)]Cl_2_, known as TLD1433) with Tf. The Tf-targeted conjugate was associated with enhanced internalization and phototoxicity in rat bladder cancer cells when compared with the non-targeted counterpart. In vivo studies with mice bearing the highly immunogenic CT26.CL25 tumors revealed approximately 70% of overall survival with the Tf-targeted conjugate (50 mg/kg, 600 J/cm^2^ at 808 nm), whereas only ≈30% was attained with the ruthenium complex alone [139,140,141].

### 3.2. Antibody and Nanobody-Targeted PSs

Antibodies and their fragments constitute another class of moieties commonly used for PS delivery which has increased in popularity with the progression of personalized medicine. Conjugation through lysine (amide and isothiocyante conjugation) or cysteine (maleimide conjugation), SNAP-Tag conjugation and “click” chemistry (copper-catalyzed alkyne-azide cycloaddition and copper-free strain-promoted alkyne-azide cycloaddition) are the most common synthetic strategies for the development of tetrapyrrole-based antibody-PS conjugates. This has been recently discussed in great detail by Sandland and Boyle [99].

One of the most promising examples of antibody-targeted PS relies on the water-soluble silica phthalocyanine-based PS IRDye700DX (IR700), which has been conjugated to different mAbs. Initially, studies performed with trastuzumab or panitumumab (anti-EGFR mAb)-targeted IR700 showed a preferential accumulation of the PS at the A31 cell membrane, inducing necrotic cell death upon illumination at 690 nm. In vivo specific A431 (epidermoid) and 3T3/HER2 (breast) tumor accumulation and shrinkage were initially reported (300 µg/mouse, DLI = 24 h, DL = 30 J/cm^2^) [142]. This strategy was further investigated for bladder cancer treatment, either in monotherapy with panitumumab-targeted IR700 [143] or upon combination of the latter with trastuzumab (anti-HER2)-targeted IR700 [144]. Additional works have demonstrated that this strategy can be effective for tumors of different histological origin by using antibodies against relevant targets. Examples include prostate cancer (prostate-specific membrane antigen, known as PSMA) [145,146]; oral cancer (CD44); lung cancer (delta-like protein 3) [147,148]; glioblastoma (CD133) [149]; and melanoma (CD146) [150].

Overall, mAb-IR700 has the ability to induce specific cell death of cancer cells while sparing adjacent healthy tissue, tumor vessels and infiltrating-immune cells [142,143,144,145,146,147,148,149,150]. The mechanism triggering necrotic cell death was recently highlighted by Sato et al. [151]. By using trastuzumab, panitumumab or cetuximab-targeted IR700, the authors showed that a light-induced ligand-release reaction occurs upon illumination at 690 nm. The latter affects the physical properties of the conjugate, inducing physical stress, which, in turn, leads to the disruption of the cell membrane, cell swelling and blebbing followed by bursting of the membrane. Importantly, this enables the release of the intracellular content (including danger associated molecular patterns, DAMPS), thereby triggering the activation of the host immune system. This systemic response contributes to the long-term control of the disease and further therapeutic improvements can be achieved through inhibition of immune checkpoint blockers. For instance, combination of cetuximab-targeted IR700 with blockade of the PD1/PLL1 axis was shown. This resulted in complete rejection of MC38 tumors and inhibition of distant (and not illuminated) metastasis [152]. Phase 1/2 clinical trials (NCT02422979) of cetuximab-IR700 (RM1929) in patients with recurrent and advanced head and neck squamous cell cancer demonstrated encouraging results with several cases of partial remissions and others of complete remission [153,154]. Currently, the phase 3 clinical trial (NCT03769506) is ongoing [155].

Work continues to explore antibody-IR700 conjugates, such as the case-study reported by Isobe et al., wherein this PS was conjugated with an antibody targeting delta-like protein 3 (a specific biomarker of small-cell lung cancer) [148]. PDT resulted in reduced growth in tumor size of small cell lung cancer (i.e., SBC3 and SBC5 cell lines) xenografts in mice. Other examples of antibody-targeted PS are revealed in the works of Aung et al. and Darwish et al. using 1849-indocyanine green and a phthalocyaninato zinc(II) (ZnPc), respectively [156,157]. Aung et al. conjugated the PS with an antibody targeting tissue factor (TF) which is overexpressed in pancreatic cells [156]. Selectivity for pancreatic tumors in vitro and in vivo, and reduced tumor growth in xenografts, were shown. Darwish et al. targeted the CD38 glycoprotein which is overexpressed in myeloma and confirmed their hypothesis of increased selectivity and phototoxicity in vivo with their novel conjugate [157].

IR700 has also been successfully targeted to different types of cancer cells by means of nanobodies. Nanobodies are small but fully-functional fragments of the classical antibodies, each consisting of only a single variable heavy chain. The single domain structure leads to not only reduced immunogenicity in targeting but enhanced tumor diffusion and a more homogenous tissue distribution in comparison to intact mAbs. Driel et al. demonstrated that EGFR nanobody-IR700 conjugate selectively accumulated, at a time point as short as 1 h, in orthotopic OSC head and neck tumors, leading to ≈90% tumor necrosis while sparing adjacent healthy tissues [105]. Heukers et al. recently published the use of a nanobody to target IR700 to cancer cells overexpressing c-Met, a receptor tyrosine kinase also known as hepatocyte growth factor receptor [158]. The nanobody-PS conjugate specifically killed gastric MK45N cancer cells in the nanomolar range. De Groof et al. reported a nanobody-IR700 conjugate to target cells expressing US28, a viral G protein-coupled receptor (GPCR) that has an oncomodulatory effect in the progression of glioblastoma [123]. The nanobody-targeted IR700 selectively destroyed glioblastoma cells in 2D and 3D in vitro cultures, showing potential for in vivo PDT. Notably, better tumor penetration and faster clearance was achieved in comparison to an anti-US28 antibody-targeted IR700 conjugate. This demonstrated the binding superiority of the nanobody in comparison to previously reported antibody [159].

Lastly, affibodies are another interesting class of targeting moieties. Affibodies are synthesized peptide mimetics of antibodies that have high specificity towards specific proteins. Owing to their small size (6–7 kDa), they exhibit better tissue penetration. Yamaguchi et al. reported the conjugation of IR700 to an affibody targeting HER2 [160]. The results pointed to a clear selectivity of the conjugate for HER2-overexpressing breast cancer cells, and a strong phototoxic effect mirrored in the low cell viability measured. The cell death mechanism observed with this affibody-PS conjugate was similar to the one proposed by Sato et al. [151].

### 3.3. Peptides-Targeted PS

The use of small peptides for the delivery of PS generally improves solubility in aqueous solutions, leading to higher phototoxicity and therapeutic efficacy [161]. Solution- or solid-phase strategies, involving carboxylic acid activations, Michael additions and Huisgen cycloadditions, have been used for conjugation of peptides to PSs and are discussed in detail by Williams et al. [162].

The peptide GE11 [39], discovered through phage display against EGFR, has attracted the interest of several researchers. Yu et al. reported the synthesis, characterization and in vitro phototoxicity of a GE11-targeted 1,4-bis(triethylene glycol)-substituted carboxyl ZnPc [161]. Enhanced internalization and phototoxicity of the GE11-PS conjugate was observed in EGFR-positive cells (A431 cells) but not in low-EGFR-expressing cells (MCF7). Biodistribution studies through in vivo fluorescence imaging revealed enhanced accumulation of the GE11-PS conjugate in A431 tumors in comparison to the PS attached to a control peptide. However, PDT efficacy in a cancer mouse model was not investigated. More recently, Kim et al. have also reported a GE11-chlorin(e_4_) conjugate (RedoxT) for theranostics [163,164]. The in vitro assays using HCC70 cells showed specific EGFR-mediated uptake and enhanced phototoxic effect of the conjugate [163]. Triple-negative breast cancer cells overexpressing EGFR (e.g., MDA-MB-231 and MDA-MB-468 cells) also benefited from this targeting approach, which was shown to be useful for in vivo near infrared (NIR) fluorescence imaging on xenograft mouse models [164]. With the aim of targeting sex-hormone-dependent tumors (namely breast cancers), a mono-substituted β-carboxyl ZnPc was targeted to gonadotropin-releasing hormone receptors (GnRHRs) upon conjugation with two GnRH peptide analogues. This included a native GnRH peptide which was directly conjugated to the PS, and an optimized form with a D-Lys as an anchoring point for the lysosomally cleavable hexapeptidic spacer (GGGFLG) which connects to the ZnPc. For the two conjugates, the selectivity and phototoxicity was higher in comparison to the free ZnPc, both in vitro and in vivo, in breast cancer models. Of note, the optimized analogue inhibits the blood–brain barrier crossing which is typically observed in GnRHR targeting. It also exhibited less skin accumulation. Thus, it might constitute a valuable targeting approach for breast cancer [165]. A correlation between higher GnRHRs expression and worse prognosis for head and neck squamous cell carcinoma (HNSCC) was also recently established, suggesting GnRHRs as potential targets for this type of cancer. In accordance, the conjugation of GnRH peptides to protoporphyrin IX was shown to effectively inhibit the viability of Detroit-562 pharyngeal carcinoma cells when compared to the free PS [166].

Recently, Zhang et al. developed an approach which envisages the targeting of the cell membrane of cancer cells without promoting cellular internalization [167]. To achieve this goal, the authors attached Pp IX to the K-Ras-derived peptide, KKKKKKSKTKC-OMe, which has the ability to target the plasma membrane. The bioconjugate was able to destroy the cellular membrane of 4T1 cells at low concentrations, allowing a fast release of DAMPs, and therefore, immunogenic cell death. Both in vitro (4T1 cell line) and in vivo (4T1 tumor-bearing mice) assays showed an increased anti-tumoral effect of the bioconjugate when compared with the non-targeted counterpart. The triggered anti-tumor immunity was strong enough to inhibit the growth of contra-lateral (and non-illuminated) tumors and was potentiated upon combination with programmed cell death receptor 1 (PD1) blockade [167].

### 3.4. Other Targeting Strategies

While the abovementioned targeting strategies are the most used, additional approaches have been investigated for the development of targeted-PS conjugates (Table 3). Along with FA and Trf, other endogenous ligands have been investigated for anticancer targeted-PDT. For instance, biotin receptors have been shown to be more overexpressed than FRα in several cancer cell lines of different histological origin (e.g., colon, breast, renal, lung and leukemia) [168]. Biotin-targeted PSs were demonstrated to promote specific and enhanced accumulation in cancer cells, which was correlated with improved phototoxicity compared to what was obtained with free forms [168,169,170,171]. The synthesis of steroid-targeted PS is also a relatively common strategy, especially when targeting hormone-dependent tumors such as breast or ovarian cancers [172,173,174].

The metabolic changes of tumors may be explored as another approach to develop targeting strategies for cancer. Due to the high demand for glucose (Warburg effect) and cholesterol by the cancer cells, conjugation of PS with sugar molecules or lipoproteins (LDL, HDL) is being successfully used to improve the selectivity and phototoxicity of PDT [131,175,176]. Lastly, aptamer-targeted strategies have shown specificity similar to that obtained with the antibodies [114,177,178]. The low immunogenicity, longer shelf-life and low production costs constitute important advantages over antibodies.

**Table 3:** Examples of ligand-targeted tetrapyrrole PSs.

Strategy	PS	Ligand	Target	Application	Ref.
Endogenous ligand	Chlorin derivatives	Biotin	Biotin receptor	In vitro: CT26 cells	[168]
Endogenous ligand	(Phthalocyaninato)zinc(II)	Biotin	Biotin receptor	In vitro: HeLa and HuH-7 cells	[169]
Endogenous ligand	Ruthenium (II) polypyridyl complex	Biotin	Biotin receptor	In vitro: A549R cells	[170]
Endogenous ligand	Silicon (IV) phthalocyanine	Biotin	Biotin receptor	In vivo: mice bearing HeLa tumors	[171]
Endogenous ligand	Pyropheophorbide *a*	17-substituted testosterone and epitestosterone	Androgen receptor	In vitro: LNCaP and PC-3 cells	[174]
Carbohydrate	H_2_TFPC (chlorin)	d-glucose	Glucose transporter	In vitro: MKN28, MKN45, HT29 and HCT116 cells; In vivo: mice bearing HT29 or HCT116 tumors	[175]
Carbohydrate	H_2_TFPC (chlorin)	d-mannose	CD206 (mannose receptor)	In vitro: MKN28, MKN45, HT29, HCT116 and M1- and M2-polarized THP-1 macrophages; In vivo: mice bearing CT26 tumors	[179]
Aptamer	Chlorin e_6_ free acid	AIR-3A (RNA aptamer)	Interleukin-6 receptor	In vitro: BaF3/gp130/IL6R/TNF cells expressing interleukin-6 receptor	[177]
Aptamer	Chlorin e_6_ free acid	AS1411 (DNA aptamer)	Nucleolin	In vitro: MCF-7, HCT 116 and SKOV-3 cells; Ex vivo: MCF-7 and HCT 116 tumours	[114]

## 4. Ligand-Targeted Nanocarriers for the Delivery of Photosensitizers

The development of delivery formulations that enable the systemic administration of a hydrophobic PS is an important aspect for its clinical translation. One promising strategy relies on the use of nanotechnology to create new drug delivery strategies [180]. The use of nanocarriers in the PDT field might not only improve PS solubility but also allow better pharmacokinetic and pharmacodynamic profiles, which would be expected to result in higher PS accumulations in tumors (either by passive or active targeting) while avoiding healthy tissues [181]. Overall, NPs have becoming increasingly popular within cancer PDT therapy as an effective means for PS delivery. Moreover, nanoparticles (NPs) can be prepared with different degrees of sophistication, allowing multifunctionality in a single system. In this regard, nanoplatforms which combine different therapeutic modalities (e.g., PDT + chemotherapy) or permit simultaneous diagnostic imaging and therapy functions (known as theranostics) have become very common.

The attachment of targeting moieties to the NPs’ surfaces is a strategy widely used to enhance tumor accumulation and the treatment specificity. However, with regard to tumor accumulation, the success of a targeted system is not straightforward, as many factors come into play. Indeed, the nanocarrier’s physicochemical properties (size, shape, charge, etc.), the type of ligand (proteins, peptides, antibodies, nanobodies, etc.), the target receptor (level of expression and ability to be internalized) and the pathophysiology of tumor (namely the blood and lymphatic vessels network) strongly impact tumor accumulation and cellular internalization [7]. For instance, Shmidt et al. developed a mechanistic model which suggests that active targeting of nanoparticles ≥50 nm does not improve tumor accumulation when compared with the non-targeted controls [182]. In accordance, a few works have demonstrated that targeted nanoparticles do not necessarily increase tumor accumulation, but instead cellular internalization [10,11].

**Figure 2:**
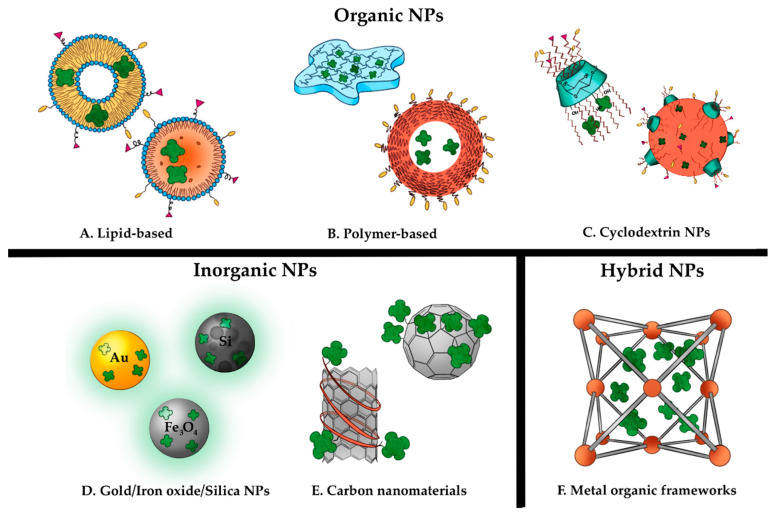
Types of nanoparticles (NPs) that have been used to improve PS (depicted by green) solubility, bioavailability and tumor targeting: (**A**) lipid-based NPs (liposomes and solid lipid NPs), (**B**) polymer-based NPs (hydrogel and PNPs), (**C**) cyclodextrin NPs, (**D**) inorganic NPs (Au, Fe and Si-NPs), (**E**) carbon nanomaterials (carbon nanotubes and fullerene) and (**F**) metal organic frameworks.

To date, many types of organic and inorganic nanoplatforms have been developed for delivery of PSs (Figure 2). Typically, organic NPs are composed of lipids or polymers with the advantages of high biocompatibility and increased PS solubility. Inorganic NPs are metallic, metal oxide and metal salt in composition and have favorable optical properties that might enhance PDT properties. In the next section, ligand-targeted NPs aimed at systemic delivery of PSs (principally tetrapyrrole PSs) for cancer treatment will be briefly discussed.

### 4.1. Ligand-Targeted Lipid-Based NPs

Lipids offer significant potential as natural and biocompatible materials for drug delivery [183]. Due to the hydrophobic nature of several PSs, lipid-based formulations have been considered as ideal carriers, which can be confirmed by the clinically approved liposomal formulation of verteporfin (known as Visudyne^®^) which is used for the treatment of age-related macular degeneration [184,185].

Liposomes are currently the most promising lipid-based NPs. Due to the development of liposomes by Bangham in 1965 [186] and thanks to evaluations of their biochemical properties over decades, liposomes have become a pioneer’s choice for drug delivery. Liposomes are typically composed of phospholipids but may also include other lipids, such as cholesterol. Typically, they can be defined as spherical vesicles having at least one (unilamellar lipid vehicles, SUV) or more (multilamellar lipid vehicles, MLV) lipid bilayers. Their structure contains two regions—hydrophilic, composed of the aqueous core, and hydrophobic, composed of lipids chains, meaning that they can be used as a drug carries for lipophilic or hydrophilic molecules [187]. Further improvements can be mediated by the functionalization of their surfaces through the chemical conjugation of targeting ligands. This is often attained via amide, disulphide or thioether bonds [188]. A variety of molecules, including mAbs (cetuximab [189], anti-HER2 [190]), peptides (cRGD [93], APRPG [191]), vitamins (biotin [192], FA [193]) and polymers (HA [194]) (Table 4) have been explored to improve liposome selectivity and cellular internalization. For instance, Kato et al. attached FA moieties to the porphyrin-conjugated lipid NPs (known as porphysomes) [195,196,197]. The presence of FA has improved the targeting ability of the formulation, resulting in enhanced cell death of lung cancer cells when compared with the non-targeted control (72% vs. 17% and 76% vs. 1% cell death in A549 and SBC5 cells, respectively). Moreover, in vivo fluorescence imaging showed specific accumulation of the Fa-targeted liposomes in A549 tumors. This correlated with a reduction of the tumor growth, a decrease of the Ki-67 cell proliferation index and enhanced cell apoptosis.

Nanocarriers offer the possibility of combining multiple therapeutic agents (PSs, chemotherapy, etc.) and other functions (ligand-targeting, imaging-mediated diagnosis, etc.) within the same system, which helps to tackle different aspects related to cancer treatment. For instance, liposomes composed of thermosensitive lipids were used to simultaneously encapsulate the chemotherapeutic drug doxorubicin (DOX) and the near-infrared photothermal (PTT) and PDT agent indocyanine green (ICG). FA and gadolinium chelates were further attached to the liposomal surface. The developed multifunctional liposomes allowed in vivo imaging by fluorescence, photoacoustic spectroscopy and magnetic resonance while tumor eradication was mediated by the combination of chemotherapy, PTT and PDT. Indeed, the FA-targeted nanoplatform caused effective phototoxicity in vitro against HeLa cells and site-specific accumulation of the FA-targeted formulation in HeLa tumors after systemic administration, which was correlated with tumor eradication for at least 2 weeks following tumor illumination [198].

An analogous approach was evaluated for a liposomal formulation combining chlorin e_6_ as PS, ICG as a PTT agent, and the hypoxia activated prodrug tirapazamine (TPZ) as a cytotoxic agent. Surface modification was employed with cRGD and conjugation of gadolinium chelates. The targeting ability of the formulation to the α_v_β_3_ integrin receptor was confirmed via intracellular fluorescence of the chlorin e_6_. Measurements confirmed enhanced cRGD-mediated endocytosis and distribution of the targeted-PS in the cytoplasm; no fluorescence signal was detected for the ligand-free formulation. Phototoxicity studies carried out in A549 lung cancer cells resulted in 97% cell death after illumination at 808/660 nm (PTT and PDT effects), and only 75% cell death was caused by illumination specifically at 808 nm (exclusively PTT effect). Further studies in A549 tumor-bearing mice revealed 5.63-fold enhanced tumor accumulation of the cRGD-targeted PS in comparison with the non-targeted control 8 h post-injection. No tumor was observed for at least 2 weeks post-treatment with the cRGD-targeted formulation and the dual-illumination at 808/660 nm, and tumor regrowth was observed for single illuminations at 808 nm. In addition, the combination of chlorin e6, gadolinium and ICG permitted a theranostic use of the formulation, by allowing in vivo fluorescence/photoacoustic/MRI imaging [93]. HA-targeted liposomes were also used for the encapsulation of ICG aimed at treated glioblastoma cancer. In vitro studies using U-87MG cells showed 65% of cancer cell death after laser radiation at 808 nm. Additional studies using nude mice bearing U-87MG tumors confirmed site-specific accumulation of the developed HA-targeted liposomes treatment was correlated with effective inhibition of tumor growth (to just 12.7% of the control group tumor size) and a decrease of the cell proliferation marker Ki-67 [199]. Despite promise, this study lacks the non-targeted control which is important to address the real impact of the HA-targeting strategy used herein. Multifunctional platforms, including those just mentioned, are attracting increasing attention, although, it is expected that this high level of sophistication may cause their clinical translation to be more challenging.

Although liposomes are the best-known lipid-based NPs, new delivery strategies based on lipids other than phospholipids have emerged in recent years. Solid lipid NPs (SLNs) are usually composed of a crystal lipophilic core (triglycerides, glyceride or waxes) which is solid at room and physiological temperatures, in which hydrophobic drugs can be encapsulated. The lipid matrix is composed of physiological lipids. Their preparation does not require the use of organic solvents and is easily scaled-up. As drug mobility decreases in the solid lipid state, drug release can be tuned by the adjustment of the composition of the solid matrix, allowing sustained release [197]. Improvements of the SLNs led to the development of nanostructured lipid carriers (NLCs), which unlike their predecessors, are formed by mixing solid and liquid lipids (oils). This results in an imperfect crystal lipid matrix that enables enhanced drug loading capacity [200]. Only a limited number of studies describing SLN/NLCs for the targeted delivery of PSs have been published. Ding et al. developed a multifunctional FA-targeted PEGylated NLC platform for chemo-PDT by combining paclitaxel (PTX) and ICG [201]. In vitro studies in human liver carcinoma cancer cells (HepG2) exhibited dose-dependent synergistic effect of PDT and chemotherapy reaching nearly 80% cell death for the highest drug concentration (2 µg/mL), while not exceeding 60% cell death for the individual ICG and PTX treatments. Treatment of HepG2 spheroids with FA-targeted formulation containing both ICG and PTX impaired their growth (<200 nm) better than NPs containing only ICG or PTX (spheroids > 300 nm). In vivo studies revealed site-specific tumor accumulation just 2 h after systemic administration. FA-targeted PEGylated NLC enabled 28.48-fold higher ICG tumor accumulation when compared to its free form and optimal ICG tumor accumulation was observed at 12 h post-injection. Other recent studies using targeted lipid-based NPs for the delivery of PSs are summarized in Table 4.

**Table 4:** Examples of ligand-targeted lipid-based NPs for photodynamic therapy (PDT).

Nanocomposition	PS	Ligand	Target	Extra Features	Application	Ref.
**Liposomes**	Erythrosine-decyl ester	Biotin	Biotin receptor	_	In vitro: ATCC^®^ CCL1.3™ cells	[192]
**Liposomes**	ICG	FA	FR	DOX, Gadolinium (III)	In vitro: HeLa, NIH-3T3 cells; In vivo: mice bearing HeLa tumors	[198]
**Liposomes**	Pyropheophorbide *a*-lipid	FA	FR	_	In vitro: A549, H647, H460, SBC5 and DFC1024 cell lines; In vivo: mice bearing A549 tumors	[196]
**Liposomes**	(5,10,15,20-Tetraporphyrinato)zinc(II)	FA	FR	_	In vitro: HeLa cells	[202]
**Liposomes**	Temoporfin	FA	FR	PEG	In vitro: A549, KB and HeLa cells	[193]
**Liposomes**	Verteporfin	Anti-EGFR antibody (Cetuximab)	EGFR	_	In vitro: Ovcar-5, CAMA-1 and A431 cells	[189]
**Liposomes**	Verteporfin	Anti-EGFR antibody (Cetuximab)	EGFR	Irinotecan	In vitro: OVCAR-5, U87 and J774 cells	[203]
**Liposomes**	Pheophorbide *a* derivative	Anti-EGFR antibody (Cetuximab)	EGFR	DOX	In vitro: A-431 SK-BR-3 cells; In vivo: A-431 tumors	[204]
**Liposomes**	Hydrophobically modified ICG with octadecylamine (ODA)	Anti-Her2 antibodies	Her2	DOX	In vitro: MCF7, SKOV3, A549 and S180 cells; In vivo: mice bearing SKOV3, A549 and MCF7 tumors	[190]
**Liposomes**	Chlorin e_6_ free acid	cRGD	α_v_β_3_ integrin receptor	TPZ, Gadolinium (III), ICG	In vitro: A549 cells In vivo: mice bearing A549 tumors	[93]
**Liposomes**	Verteporfin	Factor VII (fVII) protein	VEGFR	_	In vitro: CHO-K1, EMT6, HEK 293, MDA-MB-231 and HUVEC cells; In vivo: mice bearing EMT6 tumors	[205]
**Liposomes**	ICG	HA	CD44	PEG	In vitro: U-87MG; In vivo: mice bearing U87MG tumors	[199]
**Liposomes**	Porphyrin derivatives: 5,10,15,20-tetrakis(4-aminophenyl) porphyrin, 5, 10,15,20-tetrakis(4-hydroxyphenyl) porphyrin, 5, 10,15,20-tetraphenyl porphyrin, 5,10,15,20-tetra(4-pyridyl) porphyrin	HA	CD44	Rhodamine	In vitro: MDA-MB-231 cells	[194]
**NLC**	ICG	FA	FR	Paclitaxel, PEG	In vitro: HepG2 and NIH3T3 cells; In vivo: mice bearing HepG2 tumors	[201]
**NLC**	1,2,3,4,8,9,10,11,15,16,17,18,22,23,24,25-hexadecafluoro-29H,31H-phthalocyanine	FA	FR	_	In vitro: MCF-7 cells	[206]

### 4.2. Ligand-Targeted Polymer-Based NPs and Hydrogels

Polymeric NPs can be prepared from naturally occurring (HA [207]) or synthetic (poly lactic-co-glycolic acid (PLGA) [208], PEG [209]) polymers that exhibit good biocompatibility profiles. In polymeric NPs, depending on the preparation method, the drug can be linked to the structure in various ways, such as encapsulation, adsorption, dispersion or covalent attachment. Numerous ligand-targeted NPs of different types have been developed to improve PDT for cancer treatment and are summarized in Table 5. The most promising works with in vivo validations are discussed further herein.

PLGA is a co-polymer thermo-responsive polyester used in approved therapeutic devices and widely explored in NPs for drug delivery. It is synthesized upon ring-opening copolymerization of lactic acid (LA) and glycolic acid (GA). This has been the most popular, widely studied and most improved method for NP production over the past few decades. It allows control over the polymerization process while special attention is paid to the LA/GA ratio. The latter has a tuning impact on the hydrophobic properties of the formulation, thereby controlling the drug release kinetics [210]. Many attempts have been made to modify the surfaces of PGLA-based particles with targeting moieties to increase their specificity for cancer cells or other cells from the tumor microenvironment. Recently, two very promising approaches were reported in the literature, emphasizing their application in the field of PDT. Zhang et al. developed PGLA-based NPs that simultaneously incorporated chlorin e_6_ and DOX [211]. The Ns surface was modified with methoxy-PEG and red-blood cell (RBC) membranes to avoid immune responses, while FA was added to increase the specificity towards cancer cells. This multifunctional system resulted in 25% more HepG2 tumor accumulation and 1.3-fold higher apoptotic rates in comparison with the non-targeted formulation. Treatments with the targeted NP enabled effective suppression of tumor growth, achieving a tumor weight that was 0.51 ± 0.17 g lower when compared with tumors treated with the non-targeted approach. Of note, no pathological changes in the surrounding tissues were observed.

Chen et al. proposed cRGDfK-targeted PLGA-based NPs, aiming specifically at targeting glioblastoma cells [212]. In order to overcome the hypoxic conditions often found in the tumor site, a H_2_O_2_-activatable catalase was incorporated in the NPs. Cellular selectivity of the formulation toward α_v_β_3_ integrin was tested by comparing cellular uptake of the targeted and non-targeted formulation on α_v_β_3_ integrin overexpressed U87-MG cells. After 3 h of incubation, strong fluorescence was detected, but only for the targeted formulation, while remaining low for the control group. Phototoxicity experiments resulted in 98% cancer cell death, while only 15% was reached with the catalase-free NPs. The benefit of the cRGDfK moieties was demonstrated in mice bearing U87-MG tumors. Higher tumor accumulation and complete inhibition of tumor growth was observed for the catalase and targeting moiety containing formulation, while other approaches led to tumor growth within 8 days post-treatment.

Another promising example relies on the use of GE11-targeted PEG-polycaprolactone (PCL) NPs containing HOSiPcOSi(CH_3_)_2_-(CH_2_)_3_N(CH_3_)_2_, (Pc_4_, silicon phthalocyanine 4). The GE11-targeted NPs exhibited 15% more internalization in SCC-15 human squamous cell carcinoma cells compared to the non-targeted control. Phototoxicity in the range of 95% and reduced clonogenicity were attained with low doses of the PS (400 nM). It is important to point out that the in vivo study carried out with SCC-15 tumor bearing mice resulted in comparable tumor regression within the first 30 days of treatment for both approaches. However, unlike the GE11-targeted formulation, after that time tumor regrowth was observed in the non-targeted nanoplatform treated group [213].

Recently, hydrogels, cross-linked three-dimensional scaffolds of hydrophilic polymers with the ability to swell in aqueous media, have been gaining attention in pharmaceutical engineering [214]. Common hydrogel formulations used for drug delivery can be composed of natural (chitosan, agarose, alginates, hyaluronic acid) or synthetic (PEG, poly(*N*-isopropylacrylamide)) polymers. Hydrogels, due to their ability to swell in aqueous media, have been used in the PDT field to overcome the low solubility of PSs in polar media, while preventing the premature release of the PS without affecting its photophysical properties [215]. Moreover, depending on their composition, type of cross-linking and route of administration, hydrogels can tune the pharmacokinetic profile and biodistribution of the PS. However, it is important to note that the distinction between hydrogels and nanogels is not uniform and clearly defined. Very often the term nanogel is used to define nanosized hydrophilic polymeric materials, referring mainly to their ability to exhibit the EPR effect. Extensive research led to the development of site-specific smart gels that can be tuned to respond to physiological fluctuations (e.g., thermoresponsive hydrogels) [216] or actively target overexpressed receptors on cancer cells due to the attachment of specific ligands, such as polysaccharides [217] and antibodies [218], to the polymer surface. In the context of PDT, most of the developed hydrogels are intended for local administration followed by sustained released of the PS, which might allow one to perform multi-illumination procedures. Only a few examples of hydrogel-based NPs with proper features for systemic administration have been published.

Belali et al. developed a FA-targeted and pH-sensitive chitosan-based hydrogel conjugated with 5,10,15,20-tetrakis(4-aminophenyl)porphyrin (NH_2_-TPP) [219]. With the highest tested concentration, about 80% MCF7 human breast cancer cell death was attained, which represented an increase of two times when compared with the non-targeted formulation (40% cancer cell death). Hah et al. prepared polyacrylamide (PAA)-based hydrogel conjugated to methylene blue (MB) as the PS, and PEG chains aimed at prolonged NPs circulation in plasma [220]. The surface of the hydrogel was further decorated with the F3 peptide, which can selectively target tumor vasculature and cancer cells. Phototoxicity experiments carried out on MDA-MB-435 cells resulted in 90% cell death for the F3-targeted formulation, while the non-targeted particles only reached about 30% cell death. An analogous system was later studied using 2-devinyl-2-(1-hexyloxyethyl) pyropheophorbide (HPPH) as a PS, expanding the possible application as a theragnostic tool in cancer treatment [221]. Chitosan/alginate-based hydrogel nanoparticles were developed to improve the uptake of 5,10,15,20-tetrakis(*N*-methyl-4-pyridyl)porphyrin tetratosylate (TMP), a highly hydrophilic PS with limited ability to be internalized by cells. Antibodies targeting the death receptor 5 (DR5), which is upregulated in several types of cancer, were conjugated onto the NPs’ surfaces. In vitro studies conducted in HCT116 colorectal carcinoma cells showed two-times more cellular uptake and phototoxicity than the non-targeted control [218]. Similarly to NPs, multifunctional and stimuli-responsive hydrogels have been developed. Enzymatic-responsive hydrogels, with synergistic photodynamic (ICG) and chemotherapeutic (DOX) mechanisms of action exhibiting enhanced activity, were recently reported for head and neck cancer. Nanoparticles containing ICG or DOX were incorporated in hyaluronic acid-acrylate-based hydrogels, which were further conjugated to MMP [222]. Mice bearing SCC-15 tumors submitted to intratumoral injection of the developed multifunctional hydrogel exhibited strong tumor regression that was significantly higher than the one attained with hydrogels containing only ICG or DOX. However, the real contribution of MMP targeting is difficult to assess, as MPP free hydrogels were not tested. Despite them being encouraging, most of the described research with hydrogels lacks in vivo study, which makes an assessment of their real potential as novel pharmaceutical formulations difficult.

**Table 5:** Examples of ligand-targeted polymer-based NPs for PDT.

Nanocomposition	PS	Ligand	Target	Extra Features	Application	Ref.
**Methoxy-PEG-PLGA-based PNP**	Chlorin e_6_ free acid	FA	FR	PEG, RBC membranes, DOX	In vitro: HepG2 cells; In vivo: mice bearing HepG2 tumors	[211]
**PEGylated PLG-*co*-hydroxymethyl GA-based PNP**	*meso*-tetraphenylchlorine disulphonic acid disodium (TPCS_2a_)	anti-HER2 nanobody (11A4)	HER2	PEG, Saporin	In vitro: SkBr3 (HER2+), MDA-MB-231 (HER2-) cells	[223]
**PLGA-based PNP**	Pheophorbide *a*	FA	FR	PEG	In vitro: MKN28 cells; In vivo: mice bearing MKN28 tumors	[224]
**PLGA-based PNP**	Verteporfin	FA	FR	_	In vitro: HCT116 cells	[225]
**HA-b-PLGA-based PNP**	Pp IX	HA	CD44	_	In vitro: A549 cells	[226]
**(PLGA) and carboxymethyl chitosan (CMC)- based PNP**	Hypocrellin A	Tf	Tf receptor	_	In vitro: A549, NIH-3T3 cells; In vivo: Mice bearing A549 tumors	[227]
**PLGA-based PNP**	*meso*-tetraphenylchlorine disulphonic acid disodium (TPCS_2a_)	HA	CD44	Docetaxel	In vitro: MCF-7 and MDA-MB-231 cells	[208]
**PLGA-based PNP**	*meso*-tetraphenylchlorine disulphonic acid disodium (TPCS_2a_)	HA	CD44	Docetaxel	In vitro: MDA-MB-231 and HeLa cells	[228]
**PEG-based PNP**	Coumarin chromophore	Biotin	Biotin receptor	PEG	In vitro: HeLa cells	[208]
**1,2-distearoyl-*sn*-glycero-3-phosphoethanolamine-*N*-[maleimide(PEG-2000)-based PNP**	benzo[1,2-b:4,5-b′]dithiophene 1,1,5,5-tetraoxide	RGD-4R peptide	α_v_β_3_ integrin receptor	4,4′-(2,2-diphenylethene-1,1-diyl)bis(*N*,*N*-diphenylaniline)	In vitro: SKOV-3, HeLa, PC3 and MCF7 cells; In vivo: mice bearing SKOV-3 tumors	[229]
**PLGA- PNP**	MB	c(RGDfK) peptide	α_v_β_3_ integrin receptor	Catalase in the aqueous core, Black hole quencher-3	In vitro: U87-MG, MCF-7, SKOV-3 and HaCaT cells; In vivo: mice bearing U87-MG tumors	[212]
**PLGA-PEG-based PNP**	Verteporfin	hTf peptide	Tf receptor	_	In vitro: MDA-MB-231 cells	[139]
**PEG-PCL-based Polymeric micelles**	HOSiPcOSi(CH_3_)_2_-(CH_2_)_3_N(CH_3_)_2_, (Pc 4)	GE-11 peptide	EGFR	_	In vitro: SCC-15 cells; In vivo: mice bearing SCC-15 tumors	[213]
**Chitosan-based hydrogel**	Tetrakis(4-aminophenyl)porphyrin	FA	FR	_	In vitro: The MCF-7 (FR+) and HepG2 (FR−) cells	[219]
**Chitosan/alginate-based hydrogel**	meso-Tetra(*N*-methyl-4-pyridyl) porphine tetra tosylate (TMPyP)	Anti-DR5 antibody	Death receptor 5	_	In vitro: HCT116 cells	[218]
**HA-based hydrogel**	ICG	MMP-2	MMP-2 receptor	DOX	In vitro: SCC-15 cancer cells; In vivo: SCC-15 tumor bearing mice	[222]
**Polyacrylamide-based hydrogel**	MB	F3 peptide	Nucleolin	PEG	In vitro: MDA-MB-435 and F98 cells	[220]
**Polyacrylamide-based hydrogel**	HPPH	F3 peptide	Nucleolin	PEG	In vitro: MDA-MB-435 and 9 L cells	[221]

### 4.3. Cyclodextrin (CDs)

CDs, natural cyclic oligosaccharides, are another example of materials used for the development of nanocarriers. Natural CDs are obtained mainly by enzymatic intramolecular transglycosylation of starch and are classified as alpha (α)-CD, beta (β)-CD or gamma (γ)-CD based on their number of linked *D*-glucopyranose units (6, 7 and 8 groups, respectively) [230]. CD’s applications in therapeutics are principally due to their ability to form host–guest complexes with a broad spectrum of drug molecules. This occurs predominantly through encapsulation, covalent conjugation or non-specific external binding [231]. The use of CD-based nanocarriers permits one to improve the solubility of several drugs, including PSs. [232]. Introduction of receptor targeting moieties enhancing the anti-cancer effects of PSs, delivered by means of CD, has also been extensively studied in recent years (Table 6).

β-CDs have been widely used for drug delivery owing to their ready availability and cavity size; however, low aqueous solubility might challenge their use in parenteral administration. Despite this limitation, several works using β-CD-based NPs for the delivery of PSs have been reported. As an example, HA-targeted β-CD-based NPs, containing adamantane-modified camptothecin prodrug (via a ROS-responsive thioketal linker) and adamantane-modified THPP, were recently described. This multi-functional formulation combines PDT with light-controlled chemotherapy. Significant internalization was observed in MDA-MB-231 breast cancer cells, which overexpress CD44 receptors, but not in cells with poor expression of CD44 receptors (MCF7), thereby indicating enhanced and specific uptake of the developed HA-targeted NPs. In vitro evaluation demonstrated low cytotoxicity in the dark while high phototoxicity was observed at low doses (50 μg/mL). Of note, in vivo experiments using mice bearing MDA-MB-231 tumors showed 3.7- and 2.2-fold higher tumor inhibition comparing to free THPP and camptothecin, respectively [233]. Similarly, Yao et al. developed β-CD-based NPs combining PDT with light activable release of camptothecin (via a nitrobenzene linker) [234]. The formulation was further decorated with lactobionic acid, which allows one to target asialoglycoprotein receptors-overexpressing tumor cells. The targeted formulation exhibited potent phototoxic effect against HepG2 cancer cells, both in vitro and in vivo, when compared with the non-targeted parent. Additionally, Zhang et al. developed mannose-targeted β-CDs-based NPs containing adamantane-modified BODIPY as the PS [235]. Receptor-specific internalization and anticancer activity were confirmed in MDA-MB-231 breast cancer cells, which overexpress mannose receptors. While the mannose-targeted NPs exhibited over 90% cell death, the ligand-free NPs only caused 11% cell death. In contrast, no significant differences were observed in the phototoxicity against MCF-10A cells lacking mannose-receptor overexpression. Treatment of mice bearing MDA-MB-231 tumors showed significant tumor growth inhibition in contrast to the non-targeted formulation. Importantly, no severe adverse reactions were noted during the treatment.

**Table 6:** Examples of ligand-targeted cyclodextrin-based NPs for PDT.

Nanocomposition	PS	Ligand	Target	Extra Features	Application	Ref.
**β-cyclodextrin**	Adamantane-modified 5,10,15,20-tetrakis(4-hydroxyphenyl)-21*H*,23*H*-porphine (THPP)	HA	CD44 receptor	Adamantane-modified camptothecin prodrug	In vitro: MDA-MB-231 cells; In vivo: mice bearing MDA-MB-231 tumors	[233]
**γ-cyclodextrin**	Fullerene C_60_	FA	FR	GO	In vitro: HeLa cells	[236]
**β-cyclodextrin**	Chlorin e_6_ free acid	Adamantine-CGKRK-GFLG-EE-HAIYPRH (T7) peptide	Tf receptor	_	In vitro: MCF-7 cells	[237]
**β-cyclodextrin**	1,8-dihydroxy-3-methylanthraquinone (DHMA)	Lactobionic acid (LA)	Asialoglycoprotein receptors	PEG, camptothecin prodrug (NBCCPT),	In vitro: HepG2 cells; In vivo: mice bearing HepG2 tumors	[234]
**β-cyclodextrin**	Pheophorbide *a*	FA	FR	Adamantane	In vitro: MCF-7 and PC3 cells	[238]
**β-cyclodextrin**	Adamantane-modified BODIPY (BTA)	Mannose	Mannose receptor	Adamantane	In vitro: MDA-MB-231 and MCF-10A cells; In vivo: mice bearing MDA-MB-231 tumors	[235]
**β-cyclodextrin**	Phenanthroline modified CD-Ruthenium complex	Tf	Tf receptor	Adamantane	In vitro: A549 cells 293T cells	[239]
**β-cyclodextrin**	GO	HA	CD44	DOX, Fe_3_O_4_	In vitro: BEL-7402 cells	[240]
**β-cyclodextrin**	(Phthalocyaninato)zinc(II)	FA	FR	Camptothecin	In vitro: HEP2 cells; In vivo: mice bearing HEP2 tumors	[241]
**β-cyclodextrin**	5,10,15,20-Tetrakis(*m*-hydroxyphenyl)-21,23*H*-porphyrin (mTHPP)	Tamoxifen	Estrogen receptor	_	In vitro: MCF7 and MDA-MB-231 cells	[242]
**β-cyclodextrin**	Adamantane-modified 5-(4-carboxyphenyl)-10,15,20-triphenylporphyrin	FA	FR	DOX, GO	In vitro: HeLa and OCT-1 cells; In vivo: mice bearing HeLa tumors	[243]

### 4.4. Carbon Nanomaterials (CNMs)

CNMs are low dimensional carbon-based constructs which were introduced for the first time in the mid-1980s. The largest applications of CNMs in medicine have been found for fullerenes, graphene oxide (GO), carbon nanotubes (CNTs) and carbon dots (CDs). CNMs can be classified based on their dimensionality. 0D carbon nanomaterials include CDs and fullerenes, which can be defined as hollow cages and quasi spherical NPs, respectively. The 1D group involves cylinder-shaped CNTs, whereas graphene is a 2D structure. CNMs contain carbon atoms in *sp^2^* (usually for fullerene, GO and CNTs) or *sp^3^* (mostly for CD) hybridization that are typically arranged in hexagonal lattices. CNMs have versatile electrochemical properties which have resulted in a wide range of applications, such as sensors for diverse materials (DNA, proteins, metals, etc.). Due to their small size (usually between 1 to 100 nm), large surface area and light absorption in the NIR region, CNMs have been explored for drug delivery, including in PDT [244]. Furthermore, their ability to absorb light in the NIR region makes CNMs promising candidates for PTT, hence their use in combination therapy. However, due to poor aqueous solubility as a result of the hydrophobic interactions and unspecific accumulation in soft tissues, the use of CNMs use is limited by their potential toxicity [245]. In recent years, many attempts focusing on CNMs surface modification have been investigated, with the goal of increasing their biocompatibility and tissue specificity [246]. Among them are the attachment of hydrophilic polymers (PEG, PEI, etc.) and targeting moieties as strategies which can modify their pharmacokinetic profile, tumor accumulation and cellular internalization [247].

Significant advances in the field have been made in the last few years (Table 7). Multifunctional platforms that combine PDT with PTT, targeted delivery towards specific tumor cellular populations and other functionalities have become common. For instance, Shi et al. developed HA-targeted NCTs loaded with hematoporphyrin monomethyl ether (HMME), which combined PDT and PTT activities [248]. B16F10 cells treated with these NPs, and illumination at both 532 and 808 nm, resulted in around 90% cell death, which was significantly higher that the effect obtained with the free HMME, non-targeted NCTs or HA-targeted NCTs illuminated with a single wavelength. In contrast, no cytotoxic effect was observed in dark conditions. Treatment of B16F10 tumor-bearing mice demonstrated strong suppression of tumor growth (only 15% of the tumor volume of the control group) without systemic toxicity.

Another interesting study was provided by Zheng et al. [249]. The authors developed CD-decorated with carbon nitrite, coupled with Pp IX, PEG and the RGD peptide. Carbon nitrite was included in the formulation in order to trigger water splitting, and therefore, increased the oxygen concentration. This is of high importance, as hypoxic regions are commonly observed in solid tumors, which often compromise PDT efficacy. In vitro studies conducted in 4T1 cells demonstrated that 50% of cell death was attained with this nanoplatform in hypoxic conditions, while no photoactivity of the free PS was observed in the same conditions. In vivo biodistribution studies carried out on 4T1-tumor bearing mice showed specific tumor accumulation of the RGD-targeted CDs which was proved by monitoring increased fluorescence of the formulation in the solid tumor in comparison with other organs. This later was correlated with over 3-times stronger inhibition of tumor growth upon tumor illumination at 630 nm in comparison with free Pp IX and carbon nitride-free CD NPs, within 12 days of the experiment. Of note, anti-tumor immunity with significant impairment of distant (and not illuminated) metastasis was reported [249].

DOX loaded fullerene NPs, containing polymeric shell with tumor targeting NGR peptide were also tested against 4T1 breast cancer cells. The fullerene-based nanoplatform, with switch on/off properties, allowed for burst chemotherapeutic release after 532 nm laser irradiation. In vitro studies confirmed the impact of the peptide for enhanced targeting, resulting in 42% cell death after laser irradiation in comparison with only 20% obtained with the non-targeted counterpart. Moreover, after systemic administration, NP accumulation in the tumor area was 7.4-fold higher than that obtained with the non-targeted CDs. Reduced accumulation in heart and kidneys and decreased side effects were observed [250].

**Table 7:** Examples of ligand-targeted carbon nanomaterials for PDT.

Nanocomposition	PS	Ligand	Target	Extra Features	Application	Ref.
**CNT**	ICG	FA	FR	PTT	In vitro: HeLa cells; In vivo: mice bearing HeLa tumors	[251]
**CD**	ICG	FA	FR	Polydopamine	In vitro: HeLa cells	[252]
**CNT**	Organoselenium compound (PSeD)	AE105 polypeptide (uPAR)	Urokinase-type plasminogen activator receptor (uPAR)	pH-responsive triblock polymer composed of PEG-COOH, polyethyleneimine (PEI) and 3,4,5,6-tetrahydrophthalic anhydride (TA) (PPTA)	In vitro: MDA-MB-231 and L02 cells	[253]
**CNT**	(2-amino-phthalocyaninato)zinc(II)	FA	FR	_	In vitro: A375 cells	[254]
**CNT**	HMME	HA	CD44	_	In vitro: B16F10 cells; In vivo: Mice bearing B16F10 tumors	[248]
**CNT**	ICG	HA	CD44	_	In vitro: SCC7; In vivo: mice bearing SCC7 tumors	[255]
**GO**	ICG	Anti-epithelial cell adhesion molecule (EpCAM) antibody and A9-aptamer	PSMA	_	In vitro: LNCaP cells	[256]
**GO**	Chlorin e_6_ free acid	HA	CD44	_	In vitro: A549 cells	[257]
**GO**	Chlorin e_6_ free acid	RGD4C peptide	α_v_β_3_ integrin receptor	Polyvinylpyrrolidone (PVP)	In vitro: MGC803 cells	[258]
**GO**	Chlorin e_6_ free acid	FA	FR	_	In vitro: MGC803 cell line	[259]
**GO**	Chlorin e_6_ free acid	HA	CD44	_	In vitro: HeLa and NIH3T3 cells	[260]
**GO**	MB	FA	FR	DOX	In vitro: HeLa and MCF-7 cells	[261]
**GO**	Verteporfin	c(RGDfK) peptide	α_v_β_3_ integrin receptor	Banoxantrone dihydrochloride (AQ4N), and HIF-1α siRNA (siHIF-1α)	In vitro: Human PC-3 prostate cancer cell line; In vivo: mice bearing PC-3 tumor	[262]
**GO**	3-[1-hydroxyethyl]-3-devinyl-13^1^-β,β-dicyanomethylene-13^1^-deoxopyropheophorbide *a*	FA	FR	DOX	In vitro: Hep-G2 cells	[263]
**GO**	Chlorin e_6_ free acid	FA	FR	1, 2-Distearoyl-sn-glycero-3-phosphoethanolamine-PEG2000	In vitro: KB, A549, HeLa, HaCaT cells; In vivo: mice bearing HeLa tumors	[264]
**GO**	Pyropheophorbide *a*	Anti-integrin α_v_β_3_ antibody	α_v_β_3_ integrin receptor	_	In vitro: MCF-7, U87-MG cells	[265]
**GO**	Tetrakis(4-carboxyphenyl)porphyrin (TCPP)	FA	FR	_	In vitro: HeLa cells	[266]
**GO**	HPPH	HK peptide	α_v_β_3_ integrin receptor	PEG	In vitro: 4T1 cells; In vivo: mice bearing 4T1 tumors	[267]
**Fullerene**	Fullerene (C_60_)	FA	FR	DOX	In vitro: HeLa (FR+) and A549 and L929 (FR-) cells	[268]
**Fullerene**	Fullerene (C_60_)	FA	FR	_	In vitro: HeLa cells	[269]
**Fullerene**	Fullerene (C_60_)	HA	CD44		In vitro: HCT-116 cells; In vivo: mice bearing HCT-116 tumors	[270]
**Fullerene**	Fullerene (C_60_)	Pullulan	Asialoglycoprotein receptors (ASGPR)	_	In vitro: HepG2 cell lines; In vivo: mice bearing tumors	[271,272]
**Fullerene**	Fullerene (C_70_)	R13 Aptamer	EGFR	_	In vitro: A549 cells	[273]
**Fullerene**	Fullerene (C_60_)	D-glucosamine	GLUT-1 receptor	_	In vitro: PANC1 and PSC cells	[274]
**Fullerene**	Fullerene (C_60_)	NGR peptide	CD13/aminopeptidase N receptor	DOX, 1, 2-Distearoyl-sn-glycero-3-phosphoethanolamine -PEG	In vitro: 4T1 cells; In vivo: mice-bearing 4T1 tumors	[250]
**Fullerene**	Diadduct malonic acid-fullerene (C_60_)	NGR peptide	CD13/aminopeptidase N receptor	2-methoxyestradiol (2ME)	In vitro: MCF-7 cells	[275]
**Fullerene**	Fullerene (C_60_)	Tf	Tf receptor	HA, Artesunate	In vitro: MCF-7 cells; In vivo: mice bearing S180 tumors	[276]
**CD**	Pp IX	FA	FR		In vitro: HeLa and HT-29 cells	[277]
**CD**	CD	Heavy-chain ferritin	Tf receptor	DOX	In vitro: MCF-7 cells; In vivo: mice bearing S180 tumors	[278]
**CD**	Pp IX	RGD peptide	α_v_β_3_ integrin receptor	Carbon nitride	In vitro: MCF-7 and 4T1 cells; In vivo: mice bearing 4T1 tumors	[249]

### 4.5. Inorganic NPs

Metals and metal oxides have been introduced to the field of medicine as potential composites for electron microscopy and drug delivery [279]. The history of their application goes back to ancient times, when the therapeutic property of gold was used in the treatment of various diseases, such as epilepsy or syphilis [280]. Today, classic examples of inorganic drugs used in clinical treatment are cisplatin (an FDA approved anticancer drug) [281] and iron oxide NPs used for the treatment of glioblastoma [282]. Inorganic NPs, due to their well-defined shape and easily modified surfaces, have also been applied in the field of PDT. Silica (SiNPs), iron oxide (IONPs) and gold (AuNPs) NPs are one of the most extensively studied delivery platforms for PS [283,284]. Many efforts to improve the tumor targeting ability of inorganic NPs, while enhancing the PDT effect, were investigated (Table 8). One advantage of such NPs is their ability to encompass multiple abilities, creating multifunctional platforms. For instance, Wang et al. designed a SiNPs formulation containing the chemotherapeutic drug, DOX, and the photosensitizer, MB, which was further decorated at the surface with a nuclear localization signal peptide (KKKRK) [285]. In vitro experiments performed using a human malignant glioma cell line (U87MG) confirmed the desired targeting activity of the formulation at the highest PS concentration (500 µg/mL), showing about 70% cell death for the targeted formulation in comparison with free DOX which only exhibited 35% cell death. Moreover, in vivo studies showed site specific U87MG tumor accumulation after systemic administration of the targeted SiNPs. This was associated with five times stronger tumor ablation when compared with free DOX, and importantly, an absence of systemic toxicity [285]. In another study, multifunctional nanosystems combining 5-aminolevulinic acid (5-ALA)-PDT, PTT (AuNPs) and imaging (dye Cy7.5) properties were developed for the treatment of breast cancer. Anti-HER2 antibody and HA conjugated to the particle surface mediated an enhanced targeting ability, resulting in 2.6 times higher uptake of the SiNPs into MCF7 cells than the non-targeted approach. Furthermore, animal studies confirmed enhanced accumulation in the tumor site (12.8% accumulation ratio after systemic administration). Although high NPs accumulation was observed in other organs, such as the liver, toxicity studies did not show any signs of pathological changes or systemic inflammation [286]. IONPs are also employed in PDT. As an example, IONPs specifically designed to trigger the Fenton reaction, and decorated on their surfaces with ICG and HA, exhibited promising results for the treatment of colon cancer. In vitro studies using HCT116 and A2780 cells revealed an effective IONP internalization which was correlated with PDT/PTT-mediated cell death upon illumination at 808 nm. Total remission of HCT116 tumors was achieved with the HA-targeted IONPs upon illumination at 808 nm, which was in contrast with free ICG, empty IONPs and non-illuminated IONP-ICG-HA. In addition, these multifunctional NPs allowed in vivo photoacoustic and fluorescence imaging, which revealed the highest tumor accumulation at 6 h post injection [287].

### 4.6. Metal Organic Frameworks (MOFs)

MOFs can be defined as hybrid and crystalline constructions of metal ions clusters coordinated by multifunctional organic ligands/linkers. Due to their versatile properties regarding size, morphology, biodegradation and chemical composition, MOFs have found a very broad spectrum of applications, such as for targeted drug delivery [288,289]. A few works have used ligand-targeted MOFs in the context of PDT (Table 8). A good example of this approach relies on HA-targeted MOF NPs containing zirconium (IV)-based 5,10,15,20-tetrakis(4-carboxyphenyl)porphyrin and α-cyano-4-hydroxycinnamate. The latter is an inhibitor of monocarboxylate transporter 1, which can be used to reduce lactate uptake by cancer cells, and therefore, aerobic respirations and oxygen consumption. In vitro experiments confirmed an enhanced phototoxicity of the formulation against CT26 colon adenocarcinoma cells, in both aerobic and anaerobic conditions (cell death > 80%), while no toxicity was observed on COS7 fibroblasts. Strong inhibition of CT26 tumor growth without signs of toxicity was observed [290].

**Table 8:** Examples of ligand-targeted inorganic nanoparticles (INPs) and metal organic frameworks (MOFs) for PDT.

Nanocomposition	PS	Ligand	Target	Extra Features	Application	Ref.
**SiNPs**	MB	Nuclear localization signal peptide (KKKRK)	Nuclear receptor	DOX	In vitro: U87MG cancer cells, In vivo: U87MG tumor bearing mice	[285]
**SiNPs**	(Phthalocyaninato)zinc(II)	FA	FR	_	In vitro: A431, SCC12, CAL27 and NHEKs cells	[291]
**SiNPs**	(5-{p-[3-(2′,5′-dioxo-2′,5′-dihydro-1*H*-pyrrol-1′-yl)-*N*-3-phenoxypropyl)propanamide]-phenyl}-10,15,20-tri-p-pyridyl-porphyrine derivative	Dimannoside-carboxylate	Mannose 6-phosphate receptor	-	In vitro: LNCaP cells	[292]
**SiNPs**	Chlorin e_6_ free acid	FA	FR	FA polyethylene glycol-b-poly(asparaginyl-chidamide), DOX	In vitro: MCF-7/ADR cells	[293]
**SiNPs**	Chlorin e_6_ free acid	HA	CD44	DOX	In vitro: SCC7 cells	[294]
**SiNPs**	5,10,15,20--Tetrakis(*N*-methyl-4-pyridyl)porphyrin tetra tosylate (TMPyP4)	FA	FR	G-quadruplex DNA, DOX	In vitro: HepG2 and 3T3 cells	[295]
**SiNPs**	*N*-[3-(triethoxysilyl)propyl]-*O*-[4-(10,15,20-tri(3-hydroxyphenyl)-(2,3-dihydro)porphyrin-5-yl) phenyl]-carbamate	FA and Biotin; RGD and RAD; Cetuximab and Bovine Serum Albumin-conjugated nanoparticles		PEG	In vitro: A549, CCD-34Lu, KB cells, HeLa, A431 and HUVEC cells	[296]
**SiNPs**	5-ALA	FA	FR	_	In vitro: B16F10 cells	[297]
**SiNPs**	5-(4-carboxyphenyl)-10,15,20-triphenylchlorin (TPC)	Neuropilin-1 (NRP-1)	VEGFR	Gadolinium	In vitro: MDA-MB-23 cells; In vivo: mice bearing U87 tumors	[298]
**SiNPs**	5,10,15-Trisulphonatophenyl-20-(*N*-phenyl-*N*’-propyltriethoxysilanecarbamide)porphyrin	HA	CD44	_	In vitro: HCT-116 cells	[299]
**SiNPs**	5,10,15-Trisulphonatophenyl-20-(*N*-phenyl-*N*’-propyltriethoxysilanecarbamide)porphyrin	Mannose, galactose	Mannose, galactose receptors	Camptothecin, fluorescein isothiocyanate	In vitro: Y-79 cells	[300]
**SiNPs**	(5,10,15,20-Tetraphenylporphyrinato)palladium(II)	cRGDyK peptides	α_v_β_3_ integrin receptor	fluorescent contrast agent, ATTO647N	In vitro: MCF-7 and U87-MG cells	[301]
**SiNPs**	5,10,15-Trisulphonatophenyl-20-(*N*-phenyl-*N*’-propyltriethoxysilanecarbamide)porphyrin	Galactose	Galactose receptor	Camptothecin	In vitro: HCT-116, Capan-1 and MDA-MB-231 cells	[302]
**AuNPs**	(5,10,15,20-Tetraphenylporphyrinato)zinc(II)	FA	FR	Thioglucose	In vitro: HeLa and MCF-7 cells	[303]
**AuNPs**	ICG	RGD peptide	α_v_β_3_ integrin receptor	Doxycycline, Combretastatin A4 phosphate, PEG	In vitro: HUVEC and HT-1080 cells	[304]
**AuNPs**	Chlorin e_6_ (Ce_6_-labeled aptamer sequence)	Nucleolin-targeting aptamer AS1411	Nucleolin	DNA-programmed polymeric SNA, DOX	In vitro: HeLa cells	[305]
**AuNPs**	5-ALA	Anti-HER2 antibody, HA	HER2, CD44	PEG, Cy7.5	In vitro: MCF-7 cells; In vivo: mice bearing MCF-7 tumors	[286]
**AuNPs**	Chlorin e_6_ free acid	Anti-CD3 antibody	CIK-cells	_	In vitro: MGC-803 and GES-1 cells; In vivo: mice bearing MGC-803 tumors	[306]
**AuNPs**	HOSiPcOSi(CH_3_)_2_-(CH_2_)_3_N(CH_3_)_2_, (Pc 4)	PSMA	PSMA receptor	PEG	In vitro: PC3pip (PSMA+ ) and PC3flu (PSMA−) cells; In vivo: mice bearing PC3pip or PC3flu tumors	[307]
**AuNPs**	Chlorin e_6_ free acid	α-lipoic acid-EGF	EGFR	_	In vitro: MDA-MB-468 cells	[308]
**AuNPs**	(Phthalocyaninato)zinc(II)	Lactose-containing thiol derivative	Galectin-1 receptor		In vitro: SK-BR-3 and MDA-MB-231 cells	[309]
**AuNPs**	5-ALA	U11 peptide	Urokinase-type plasminogen activator receptor (uPAR)	CTSE-sensitive imaging agent, PEG	In vitro: PANC1-CSTE cells; In vivo: mice bearing PANC1-CSTE tumors	[310]
**AuNPs**	(5-[4-(11-mercaptoundecyloxy)phenyl]-10,15,20-triphenylporphyrin	Anti-erbB2 ICR55 antibody	ErbB2	Thiolated carboxyl terminated PEG	In vitro: SK-BR-3 cells	[311]
**AuNPs**	5-ALA	R8-PLGLAG-EK10 peptide	MMP-2	_	In vitro: SCC-7cells; In vivo: mice bearing SCC-7 tumors	[312]
**AuNPs**	Verteporfin	FA	FR	PEG-P(Asp-Hyd)-DHLA block copolymer	In vitro: HeLA cells	[313]
**AuNPs**	HOSiPcOSi(CH_3_)_2_-(CH_2_)_3_N(CH_3_)_2_, Pc 4	EGF, Tf	EGFR, Tf receptor	_	In vitro: U87-MG and LN229 cells; In vivo: mice bearing U87-MG tumors	[314]
**Au nanoclusters**	Pp IX	FA	FR	Lipoic acid	In vitro: L929 and C6 cells; In vivo: mice bearing C6 tumors	[315]
**AuNPs**	HOSiPcOSi(CH_3_)_2_-(CH_2_)_3_N(CH_3_)_2_, (Pc 4)	EGF	EGFR	_	In vitro: 9L.E29 cells; In vivo: mice bearing 9L.E29 tumors	[37]
**AuNPs**	HOSiPcOSi(CH_3_)_2_-(CH_2_)_3_N(CH_3_)_2_, (Pc 4)	Tf	Tf receptor	_	In vitro: LN229 and U87 cells; In vivo: mice bearing U87 tumors	[140]
**AuNPs**	(Phthalocyaninato)zinc(II)	Jacalin (lectin)	T antigen	Thiol-functionalized PEG	In vitro: HT-29 cells	[316]
**IONPs**	ICG	HA	CD44	amino PEG	In vitro: A2780 and HCT-116 cells; In vivo: mice bearing HCT-116 tumors	[287]
**IONPs**	5, 10, 15, 20-tetra(phenyl-4-N-met32hyl-4-pyridyl)porphyrin	AS1411 aptamer	Nucleolin	Daunomycin	In vitro: A549 and C26 cells	[317]
**IONPs**	Chlorin e_6_ free acid	HA	CD44	_	In vitro: B16F1 cells	[318]
**IONPs**	Hypericin	Lactose	Asialoglycoprotein receptors (ASGP-R)	Polydopamine	In vitro: HepG2 and MCF-7 cells	[319]
**IONPs**	Pheophorbide *a*	FA	FR32	PEG, Caffeic Acid	In vitro: MDA-MB-231 NIH3T3 and MCF-7 cells	[320]
**IONPs**	HOSiPcOSi(CH_3_)_2_-(CH_2_)_3_N(CH_3_)_2_, (Pc 4)	Fibronectin-mimetic peptide (Fmp)	Integrin β1	_	In vitro: HNSCC, M4E, 686LN and TU212 cells; In vivo: mice bearing M4E tumors	[321]
**MOF**	Tetrakis(4-carboxyphenyl)porphyrin (TCCP)	HA	CD44	CHC	In vitro: CT26, 4T1, HeLa, COS7, MCF-7 and HepG2 cells; In vivo: mice bearing CT26 tumors	[291]
**MOF**	TCPP	HA	CD44	HIF signaling inhibitor (ACF), Zirconium ions	In vitro: H22 and NIH3T3 cells; In vivo: mice bearing H22 tumors	[322]
**MOF**	(Phthalocyaninato)zinc(II)	FA	FR	DOX	In vitro: HeLa cells	[323]
**MOF**	Al(III) phthalocyanine chloride tetrasulfonic acid (AlPcS4)	Catalase (CAT) protein molecules	Cancer cell membrane antigens	Cancer cell membrane	In vitro: HeLa, COS7; In vivo: mice bearing HeLa tumors	[324]
**MOF**	TCPP	FA	FR	TPP	In vitro: SMMC-7721 cells	[325]
**MOF**	MB	cRGD	α_v_β_3_ integrin receptor	_	In vitro: A549 and HeLa cells	[326]
**MOF**	TCPP	Bovine Serum Albumin-sulfonamides (SAs) complexes	Carbonic anhydrase IX	_	In vitro: 4T1 cells; In vivo: mice bearing 4T1 tumors	[327]
**MOF**	TCPP	Sulfadiazines	Carbonic anhydrase IX	Bovine serum albumin, MnO2	In vitro: 4T1 cells; In vivo: mice bearing 4T1 tumors	[328]
**MOF**	TCPP	Aptamer of A549 lung cancer cells	A549 lung cancer cells	DOX	In vitro: A549, MCF-7 and LO2 cells	[329]

## 5. Conclusions and Future Perspectives

Tumor selectivity is widely regarded as an essential consideration in the development of any new cancer treatment and PDT is no exception. Indeed, the clinical application of PDT is often limited by the PS’s inability to preferentially accumulate in the tumor. As a consequence, side effects, such as severe skin photosensitivity, may be developed, ultimately leading to reduced quality of life for the patient. As discussed in this review, targeting ligands that are recognized and internalized by receptors overexpressed on cancer cells and/or other cells of the tumor microenvironment are being used as an attempt to enhance tumor selectivity. Despite promising, this strategy often fails to meet full expectations. This is likely explained by the high dependence of active targeting (like passive targeting) on the EPR effect to effectively reach the tumor cells. Additionally, the use of standard 2D monolayer cell cultures for in vitro testing of active targeting strategies may provide a limited view of in vivo potential. Thus, 3D cell cultures that better mimic the tumor microenvironment complexity might be a valuable tool to be explored when bridging results from 2D cell culture and in vivo experiments [330]. In addition, the frequent use of strategies with increased complexity might complicate pharmaceutical development and scale-up, making the translation from bench top to bedside a daunting task.

Despite these limitations, active targeting continues to be a promising and advantageous approach to promote effective internalization of the PSs into the targeted tumors cells. Thus, to realize the full potential of active targeting, research efforts must continue towards the development of new and improved targeting ligands with multi-targeting abilities and trans-tissue transport abilities. Along with better formulations for systemic administration of PSs, such improvements are expected to promote specific and enhanced PS tumor accumulation accompanied with internalization by different cells of the tumor microenvironment.

## Figures and Tables

**Table 2:** Ligands for active targeting.

Ligand Type	Examples	Characteristics	Advantages	Disadvantages	Ref.
**Proteins**	Transferrin	Glycoprotein Aids iron transport via TfR1	High affinity/specificity of TfR1 interaction	Potential off-target toxicity with high doses Potential competitive binding to malignant cell receptors	[33,34,85,86,87,88]
**Peptides**	RGD, Lyp-1, GE11, F3	Low molecular weight Typically <50 aas	High target receptor affinity/specificity Enhanced tumor diffusion Biocompatibility Low manufacture costs Ease of conjugation	Slow receptor identification Low stability in vivo which may be improved by chemical modifications.	[27,89,90,91,92,93,94,95]
**Antibodies**	Trastuzumab, Cetuximab	Y shaped macromolecules	High receptor target affinity/specificity Stability in vivo	Potential immunogenicity Heterogenous tumor antigen expression High cost/resource intensive production Large size limits tumor penetration	[7,30,96,97,98,99,100]
**Nanobodies**	7D12, 7D12-9G8	Small/fully functional antibody fragment	High receptor target affinity/specificity High tissue penetration High thermal and chemical stability Reduced immunogenicity relative to mAbs	Small size can lead to unfavorably high blood clearance rate which may be avoided by chemical modification	[97,101,102,103,104,105]
**Non-protein**	Folate, Polysaccharides–Hyaluronic acid (HA) Bile acids (BAs)	Folate is used for purine and pyrimidine biosynthesis HA is a component of the extracellular matrix BAs facilitate targeting of apical sodium dependent bile acid transporter (ASB)	High affinity Minimal immunogenicity	Folate conjugates may undergo slow release HA may cause off-target effects	[35,106,107,108,109,110,111]
**Aptamers**	A10 PSMA AS1411	ss-DNA/RNA Fold into distinct secondary/tertiary structures.	High target receptor affinity Minimal immunogenicity Low manufacturing cost Suitable for large scale production High thermal and chemical stability	Off-target effects may result in toxicity Susceptible to nuclease degradation in vivo if unmodified	[112,113,114,115]

## References

[1] Nichols J.W., Bae Y.H. (2014). EPR: Evidence and fallacy. J. Control. Release.

[2] Allen T.M. (2002). Ligand-targeted therapeutics in anticancer therapy. Nat. Rev. Cancer.

[3] Elkhodiry M.A., Momah C.C., Suwaidi S.R., Gadalla D., Martins A.M., Vitor R.F., Husseini G.A. (2016). Synergistic Nanomedicine: Passive, Active, and Ultrasound-Triggered Drug Delivery in Cancer Treatment. J. Nanosci. Nanotechnol..

[4] Vasir J.K., Labhasetwar V. (2005). Targeted Drug Delivery in Cancer Therapy. Technol. Cancer Res. Treat..

[5] Zheng Y., Li Z., Chen H., Gao Y. (2020). Nanoparticle-based drug delivery systems for controllable photodynamic cancer therapy. Eur. J. Pharm. Sci..

[6] Matsumura Y., Maeda H. (1986). A New Concept for Macromolecular Therapeutics in Cancer Chemotherapy: Mechanism of Tumoritropic Accumulation of Proteins and the Antitumor Agent Smancs. Cancer Res..

[7] Lin L., Xiong L., Wen Y., Lei S., Deng X., Liu Z., Chen W., Miao X. (2015). Active Targeting of Nano-Photosensitizer Delivery Systems for Photodynamic Therapy of Cancer Stem Cells. J. Biomed. Nanotechnol..

[8] Zhu G., Niu G., Chen X. (2015). Aptamer–Drug Conjugates. Bioconjug. Chem..

[9] Trapani G., Denora N., Trapani A., Laquintana V. (2012). Recent advances in ligand targeted therapy. J. Drug Target..

[10] Bartlett D.W., Su H., Hildebrandt I.J., Weber W.A., Davis M.E. (2007). Impact of tumor-specific targeting on the biodistribution and efficacy of siRNA nanoparticles measured by multimodality in vivo imaging. Proc. Natl. Acad. Sci. USA.

[11] Kirpotin D.B., Drummond D.C., Shao Y., Shalaby M.R., Hong K., Nielsen U.B., Marks J.D., Benz C.C., Park J.W. (2006). Antibody Targeting of Long-Circulating Lipidic Nanoparticles Does Not Increase Tumor Localization but Does Increase Internalization in Animal Models. Cancer Res..

[12] David A. (2017). Peptide ligand-modified nanomedicines for targeting cells at the tumor microenvironment. Adv. Drug Deliv. Rev..

[13] Jain M., Venkatraman G., Batra S.K. (2007). Optimization of Radioimmunotherapy of Solid Tumors: Biological Impediments and Their Modulation. Clin. Cancer Res..

[14] Gosk S., Moos T., Gottstein C., Bendas G. (2008). VCAM-1 directed immunoliposomes selectively target tumor vasculature in vivo. Biochim. Biophys. Acta BBA Biomembr..

[15] Kwiatkowski S., Knap B., Przystupski D., Saczko J., Kędzierska E., Knap-Czop K., Kotlińska J., Michel O., Kotowski K., Kulbacka J. (2018). Photodynamic therapy – mechanisms, photosensitizers and combinations. Biomed. Pharmacother..

[16] Callaghan S., Senge M.O. (2018). The good, the bad, and the ugly – controlling singlet oxygen through design of photosensitizers and delivery systems for photodynamic therapy. Photochem. Photobiol. Sci..

[17] Weishaupt K.R., Gomer C.J., Dougherty T.J. (1976). Identification of Singlet Oxygen as the Cytotoxic Agent in Photo-inactivation of a Murine Tumor. Cancer Res..

[18] Zhang J., Jiang C., Figueiró Longo J.P., Azevedo R.B., Zhang H., Muehlmann L.A. (2018). An updated overview on the development of new photosensitizers for anticancer photodynamic therapy. Acta Pharm. Sin. B.

[19] Dąbrowski J.M., Arnaut L.G. (2015). Photodynamic therapy (PDT) of cancer: From local to systemic treatment. Photochem. Photobiol. Sci..

[20] Lucky S.S., Soo K.C., Zhang Y. (2015). Nanoparticles in Photodynamic Therapy. Chem. Rev..

[21] Moserova I., Kralova J. (2012). Role of ER Stress Response in Photodynamic Therapy: ROS Generated in Different Subcellular Compartments Trigger Diverse Cell Death Pathways. PLoS ONE.

[22] Donohoe C., Senge M.O., Arnaut L.G., Gomes-da-Silva L.C. (2019). Cell death in photodynamic therapy: From oxidative stress to anti-tumor immunity. Biochim. Biophys. Acta BBA Rev. Cancer.

[23] Kessel D. (2019). Photodynamic Therapy: A Brief History. J. Clin. Med..

[24] Detty M.R., Gibson S.L., Wagner S.J. (2004). Current Clinical and Preclinical Photosensitizers for Use in Photodynamic Therapy. J. Med. Chem..

[25] Li X., Lee S., Yoon J. (2018). Supramolecular photosensitizers rejuvenate photodynamic therapy. Chem. Soc. Rev..

[26] Polo L., Valduga G., Jori G., Reddi E. (2002). Low-density lipoprotein receptors in the uptake of tumour photosensitizers by human and rat transformed fibroblasts. Int. J. Biochem. Cell Biol..

[27] Chitgupi U., Qin Y., Lovell J.F. (2017). Targeted Nanomaterials for Phototherapy. Nanotheranostics.

[28] Hanahan D., Coussens L.M. (2012). Accessories to the Crime: Functions of Cells Recruited to the Tumor Microenvironment. Cancer Cell.

[29] Quail D.F., Joyce J.A. (2013). Microenvironmental regulation of tumor progression and metastasis. Nat. Med..

[30] Das M., Mohanty C., Sahoo S.K. (2009). Ligand-based targeted therapy for cancer tissue. Expert Opin. Drug Deliv..

[31] Cheung A., Bax H.J., Josephs D.H., Ilieva K.M., Pellizzari G., Opzoomer J., Bloomfield J., Fittall M., Grigoriadis A., Figini M. (2016). Targeting folate receptor alpha for cancer treatment. Oncotarget.

[32] Qian Z.M., Li H., Sun H., Ho K. (2002). Targeted Drug Delivery via the Transferrin Receptor-Mediated Endocytosis Pathway. Pharmacol. Rev..

[33] Daniels T.R., Bernabeu E., Rodríguez J.A., Patel S., Kozman M., Chiappetta D.A., Holler E., Ljubimova J.Y., Helguera G., Penichet M.L. (2012). The transferrin receptor and the targeted delivery of therapeutic agents against cancer. Biochim. Biophys. Acta.

[34] Alexander-Bryant A.A., Vanden Berg-Foels W.S., Wen X., Tew K.D., Fisher P.B. (2013). Chapter One—Bioengineering Strategies for Designing Targeted Cancer Therapies. Advances in Cancer Research.

[35] Bazak R., Houri M., El Achy S., Kamel S., Refaat T. (2015). Cancer active targeting by nanoparticles: A comprehensive review of literature. J. Cancer Res. Clin. Oncol..

[36] Danhier F., Feron O., Préat V. (2010). To exploit the tumor microenvironment: Passive and active tumor targeting of nanocarriers for anti-cancer drug delivery. J. Control. Release.

[37] Meyers J.D., Cheng Y., Broome A.-M., Agnes R.S., Schluchter M.D., Margevicius S., Wang X., Kenney M.E., Burda C., Basilion J.P. (2015). Peptide-Targeted Gold Nanoparticles for Photodynamic Therapy of Brain Cancer. Part. Part. Syst. Charact..

[38] Schneider M.R., Wolf E. (2009). The epidermal growth factor receptor ligands at a glance. J. Cell. Physiol..

[39] Li Z., Zhao R., Wu X., Sun Y., Yao M., Li J., Xu Y., Gu J. (2005). Identification and characterization of a novel peptide ligand of epidermal growth factor receptor for targeted delivery of therapeutics. FASEB J..

[40] Masoud V., Pagès G. (2017). Targeted therapies in breast cancer: New challenges to fight against resistance. World J. Clin. Oncol..

[41] Spector N.L., Blackwell K.L. (2009). Understanding the Mechanisms Behind Trastuzumab Therapy for Human Epidermal Growth Factor Receptor 2–Positive Breast Cancer. J. Clin. Oncol..

[42] Al-Hajj M., Wicha M.S., Benito-Hernandez A., Morrison S.J., Clarke M.F. (2003). Prospective identification of tumorigenic breast cancer cells. Proc. Natl. Acad. Sci. USA.

[43] Boiko A.D., Razorenova O.V., van de Rijn M., Swetter S.M., Johnson D.L., Ly D.P., Butler P.D., Yang G.P., Joshua B., Kaplan M.J. (2010). Human melanoma-initiating cells express neural crest nerve growth factor receptor CD271. Nature.

[44] Yu Z., Pestell T.G., Lisanti M.P., Pestell R.G. (2012). Cancer stem cells. Int. J. Biochem. Cell Biol..

[45] Hu Z. (2018). Therapeutic Antibody-Like Immunoconjugates against Tissue Factor with the Potential to Treat Angiogenesis-Dependent as Well as Macrophage-Associated Human Diseases. Antibodies.

[46] Chen C., Zhao S., Karnad A., Freeman J.W. (2018). The biology and role of CD44 in cancer progression: Therapeutic implications. J. Hematol. Oncol..

[47] Zhang S., Wu C.C.N., Fecteau J.-F., Cui B., Chen L., Zhang L., Wu R., Rassenti L., Lao F., Weigand S. (2013). Targeting chronic lymphocytic leukemia cells with a humanized monoclonal antibody specific for CD44. Proc. Natl. Acad. Sci. USA.

[48] Yang L., Shi P., Zhao G., Xu J., Peng W., Zhang J., Zhang G., Wang X., Dong Z., Chen F. (2020). Targeting cancer stem cell pathways for cancer therapy. Signal Transduct. Target. Ther..

[49] Hanahan D., Weinberg R.A. (2011). Hallmarks of Cancer: The Next Generation. Cell.

[50] Papetti M., Herman I.M. (2002). Mechanisms of normal and tumor-derived angiogenesis. Am. J. Physiol. Cell Physiol..

[51] Senge M.O., Radomski M.W. (2013). Platelets, photosensitizers, and PDT. Photodiagn. Photodyn. Ther..

[52] Seebacher N.A., Stacy A.E., Porter G.M., Merlot A.M. (2019). Clinical development of targeted and immune based anti-cancer therapies. J. Exp. Clin. Cancer Res..

[53] Hamidi H., Pietilä M., Ivaska J. (2016). The complexity of integrins in cancer and new scopes for therapeutic targeting. Br. J. Cancer.

[54] Jiang Y., Dai J., Yao Z., Shelley G., Keller E.T. (2017). Abituzumab Targeting of αV-Class Integrins Inhibits Prostate Cancer Progression. Mol. Cancer Res..

[55] Wagner S., Rothweiler F., Anhorn M.G., Sauer D., Riemann I., Weiss E.C., Katsen-Globa A., Michaelis M., Cinatl J., Schwartz D. (2010). Enhanced drug targeting by attachment of an anti αv integrin antibody to doxorubicin loaded human serum albumin nanoparticles. Biomaterials.

[56] Élez E., Kocáková I., Höhler T., Martens U.M., Bokemeyer C., Van Cutsem E., Melichar B., Smakal M., Cso˝szi T., Topuzov E. (2014). 507PD—Poseidon Phase I/II Trial: Abituzumab Combined with Cetuximab Plus Irinotecan As Second-Line Treatment for Patients with Kras Wild-Type Metastatic Colorectal Cancer. Ann. Oncol..

[57] Raab-Westphal S., Marshall J.F., Goodman S.L. (2017). Integrins as Therapeutic Targets: Successes and Cancers. Cancers.

[58] Sharma R., Sharma R., Khaket T.P., Dutta C., Chakraborty B., Mukherjee T.K. (2017). Breast cancer metastasis: Putative therapeutic role of vascular cell adhesion molecule-1. Cell. Oncol..

[59] Byrne J.D., Betancourt T., Brannon-Peppas L. (2008). Active targeting schemes for nanoparticle systems in cancer therapeutics. Adv. Drug Deliv. Rev..

[60] Schlesinger M., Bendas G. (2015). Vascular cell adhesion molecule-1 (VCAM-1)—An increasing insight into its role in tumorigenicity and metastasis. Int. J. Cancer.

[61] Chang A.-C., Chen P.-C., Lin Y.-F., Su C.-M., Liu J.-F., Lin T.-H., Chuang S.-M., Tang C.-H. (2018). Osteoblast-secreted WISP-1 promotes adherence of prostate cancer cells to bone via the VCAM-1/integrin α_4_β_1_ system. Cancer Lett..

[62] Liu C., Yang Y., Chen L., Lin Y.-L., Li F. (2014). A Unified Mechanism for Aminopeptidase *N*-based Tumor Cell Motility and Tumor-homing Therapy. J. Biol. Chem..

[63] Saiki I., Yoneda J., Azuma I., Fujii H., Abe F., Nakajima M., Tsuruo T. (1993). Role of aminopeptidase N (CD13) in tumor-cell invasion and extracellular matrix degradation. Int. J. Cancer.

[64] Pasqualini R., Koivunen E., Kain R., Lahdenranta J., Sakamoto M., Stryhn A., Ashmun R.A., Shapiro L.H., Arap W., Ruoslahti E. (2000). Aminopeptidase N is a receptor for tumor-homing peptides and a target for inhibiting angiogenesis. Cancer Res..

[65] Laakkonen P., Porkka K., Hoffman J.A., Ruoslahti E. (2002). A tumor-homing peptide with a targeting specificity related to lymphatic vessels. Nat. Med..

[66] Fogal V., Zhang L., Krajewski S., Ruoslahti E. (2008). Mitochondrial/Cell-Surface Protein p32/gC1qR as a Molecular Target in Tumor Cells and Tumor Stroma. Cancer Res..

[67] Fidler I.J. (2003). The pathogenesis of cancer metastasis: The “seed and soil” hypothesis revisited. Nat. Rev. Cancer.

[68] Seyfried T.N., Huysentruyt L.C. (2013). On the origin of cancer metastasis. Crit. Rev. Oncogen..

[69] Laakkonen P., Akerman M.E., Biliran H., Yang M., Ferrer F., Karpanen T., Hoffman R.M., Ruoslahti E. (2004). Antitumor activity of a homing peptide that targets tumor lymphatics and tumor cells. Proc. Nat. Acad. Sci. USA.

[70] Paasonen L., Sharma S., Braun G.B., Kotamraju V.R., Chung T.D.Y., She Z.-G., Sugahara K.N., Yliperttula M., Wu B., Pellecchia M. (2016). New p32/gC1qR Ligands for Targeted Tumor Drug Delivery. ChemBioChem.

[71] Lam P.Y.H., Hillyar C.R.T., Able S., Vallis K.A. (2016). Synthesis and evaluation of an 18F-labeled derivative of F3 for targeting surface-expressed nucleolin in cancer and tumor endothelial cells. J. Label. Comp. Radiopharm..

[72] Fonseca N.A., Rodrigues A.S., Rodrigues-Santos P., Alves V., Gregório A.C., Valério-Fernandes Â., Gomes-da-Silva L.C., Rosa M.S., Moura V., Ramalho-Santos J. (2015). Nucleolin overexpression in breast cancer cell sub-populations with different stem-like phenotype enables targeted intracellular delivery of synergistic drug combination. Biomaterials.

[73] Porkka K., Laakkonen P., Hoffman J.A., Bernasconi M., Ruoslahti E. (2002). A fragment of the HMGN2 protein homes to the nuclei of tumor cells and tumor endothelial cells in vivo. Proc. Natl. Acad. Sci. USA.

[74] Gomes-da-Silva L.C., Santos A.O., Bimbo L.M., Moura V., Ramalho J.S., Pedroso de Lima M.C., Simões S., Moreira J.N. (2012). Toward a siRNA-containing nanoparticle targeted to breast cancer cells and the tumor microenvironment. Int. J. Pharm..

[75] Gomes-da-Silva L.C., Fernández Y., Abasolo I., Schwartz S., Ramalho J.S., Pedroso de Lima M.C., Simões S., Moreira J.N. (2013). Efficient intracellular delivery of siRNA with a safe multitargeted lipid-based nanoplatform. Nanomedicine.

[76] Gomes-da-Silva L.C., Ramalho J.S., Pedroso de Lima M.C., Simões S., Moreira J.N. (2013). Impact of anti-PLK1 siRNA-containing F3-targeted liposomes on the viability of both cancer and endothelial cells. Eur. J. Pharm. Biopharm..

[77] Sugahara K.N., Teesalu T., Karmali P.P., Kotamraju V.R., Agemy L., Girard O.M., Hanahan D., Mattrey R.F., Ruoslahti E. (2009). Tissue-Penetrating Delivery of Compounds and Nanoparticles into Tumors. Cancer Cell.

[78] Teesalu T., Sugahara K.N., Kotamraju V.R., Ruoslahti E. (2009). C-end rule peptides mediate neuropilin-1-dependent cell, vascular, and tissue penetration. Proc. Natl. Acad. Sci. USA.

[79] Park J.-H., von Maltzahn G., Xu M.J., Fogal V., Kotamraju V.R., Ruoslahti E., Bhatia S.N., Sailor M.J. (2010). Cooperative nanomaterial system to sensitize, target, and treat tumors. Proc. Natl. Acad. Sci. USA.

[80] Christian S., Pilch J., Akerman M.E., Porkka K., Laakkonen P., Ruoslahti E. (2003). Nucleolin expressed at the cell surface is a marker of endothelial cells in angiogenic blood vessels. J. Cell. Biol..

[81] Winer I., Wang S., Lee Y.-E.K., Fan W., Gong Y., Burgos-Ojeda D., Spahlinger G., Kopelman R., Buckanovich R.J. (2010). F3-Targeted Cisplatin-Hydrogel Nanoparticles as an Effective Therapeutic That Targets Both Murine and Human Ovarian Tumor Endothelial Cells In vivo. Cancer Res..

[82] Liu X., Jiang J., Ji Y., Lu J., Chan R., Meng H. (2017). Targeted drug delivery using iRGD peptide for solid cancer treatment. Mol. Syst. Des. Eng..

[83] Sharma S., Kotamraju V.R., Mölder T., Tobi A., Teesalu T., Ruoslahti E. (2017). Tumor-Penetrating Nanosystem Strongly Suppresses Breast Tumor Growth. Nano Lett..

[84] An P., Lei H., Zhang J., Song S., He L., Jin G., Liu X., Wu J., Meng L., Liu M. (2004). Suppression of tumor growth and metastasis by a VEGFR-1 antagonizing peptide identified from a phage display library. Int. J. Cancer.

[85] O’Keefe D.O., Draper R.K. (1985). Characterization of a transferrin-diphtheria toxin conjugate. J. Biol. Chem..

[86] Cavanaugh P.G. (2002). Synthesis of Chlorin e6-Transferrin and Demonstration of Its Light-Dependent in vitro Breast Cancer Cell Killing Ability. Breast Cancer Res. Treat..

[87] Liu K., Dai L., Li C., Liu J., Wang L., Lei J. (2016). Self-assembled targeted nanoparticles based on transferrin-modified eight-arm-polyethylene glycol–dihydroartemisinin conjugate. Sci. Rep..

[88] Singh R.P., Sharma G., Sonali, Agrawal P., Pandey B.L., Koch B., Muthu M.S. (2016). Transferrin receptor targeted PLA-TPGS micelles improved efficacy and safety in docetaxel delivery. Int. J. Biol. Macromol..

[89] Zhong Y., Meng F., Deng C., Zhong Z. (2014). Ligand-Directed Active Tumor-Targeting Polymeric Nanoparticles for Cancer Chemotherapy. Biomacromolecules.

[90] Li Z.J., Cho C.H. (2012). Peptides as targeting probes against tumor vasculature for diagnosis and drug delivery. J. Transl. Med..

[91] Brown K.C. (2010). Peptidic tumor targeting agents: The road from phage display peptide selections to clinical applications. Curr. Pharm. Des..

[92] Dubey P.K., Mishra V., Jain S., Mahor S., Vyas S.P. (2004). Liposomes Modified with Cyclic RGD Peptide for Tumor Targeting. J. Drug Target..

[93] Dai Y., Wang B., Sun Z., Cheng J., Zhao H., Wu K., Sun P., Shen Q., Li M., Fan Q. (2019). Multifunctional Theranostic Liposomes Loaded with a Hypoxia-Activated Prodrug for Cascade-Activated Tumor Selective Combination Therapy. ACS Appl. Mater. Interfaces.

[94] Mansur A.A.P., Carvalho S.M., Lobato Z.I.P., de FáTIMA Leite M., da Silva Cunha A., Mansur H.S. (2018). Design and Development of Polysaccharide-Doxorubicin-Peptide Bioconjugates for Dual Synergistic Effects of Integrin-Targeted and Cell-Penetrating Peptides for Cancer Chemotherapy. Bioconjug. Chem..

[95] Hou J., Diao Y., Li W., Yang Z., Zhang L., Chen Z., Wu Y. (2016). RGD peptide conjugation results in enhanced antitumor activity of PD0325901 against glioblastoma by both tumor-targeting delivery and combination therapy. Int. J. Pharm..

[96] Schrama D., Reisfeld R.A., Becker J.C. (2006). Antibody targeted drugs as cancer therapeutics. Nat. Rev. Drug Discov..

[97] Kijanka M., Dorresteijn B., Oliveira S., van Bergen en Henegouwen P.M. (2015). Nanobody-based cancer therapy of solid tumors. Nanomedicine.

[98] Lewis Phillips G.D., Li G., Dugger D.L., Crocker L.M., Parsons K.L., Mai E., Blättler W.A., Lambert J.M., Chari R.V.J., Lutz R.J. (2008). Targeting HER2-Positive Breast Cancer with Trastuzumab-DM1, an Antibody–Cytotoxic Drug Conjugate. Cancer Res..

[99] Sandland J., Boyle R.W. (2019). Photosensitizer Antibody–Drug Conjugates: Past, Present, and Future. Bioconjug. Chem..

[100] Chen F., Hong H., Zhang Y., Valdovinos H.F., Shi S., Kwon G.S., Theuer C.P., Barnhart T.E., Cai W. (2013). In Vivo Tumor Targeting and Image-Guided Drug Delivery with Antibody-Conjugated, Radiolabeled Mesoporous Silica Nanoparticles. ACS Nano.

[101] Marques A.C., Costa P.J., Velho S., Amaral M.H. (2020). Functionalizing nanoparticles with cancer-targeting antibodies: A comparison of strategies. J. Control. Release.

[102] Wu A.M., Senter P.D. (2005). Arming antibodies: Prospects and challenges for immunoconjugates. Nat. Biotechnol..

[103] Sadeqzadeh E., Rahbarizadeh F., Ahmadvand D., Rasaee M.J., Parhamifar L., Moghimi S.M. (2011). Combined MUC1-specific nanobody-tagged PEG-polyethylenimine polyplex targeting and transcriptional targeting of tBid transgene for directed killing of MUC1 over-expressing tumour cells. J. Control. Release.

[104] Deken M.M., Kijanka M.M., Beltrán Hernández I., Slooter M.D., de Bruijn H.S., van Diest P.J., van Bergen en Henegouwen P.M.P., Lowik C.W.G.M., Robinson D.J., Vahrmeijer A.L. (2020). Nanobody-targeted photodynamic therapy induces significant tumor regression of trastuzumab-resistant HER2-positive breast cancer, after a single treatment session. J. Control. Release.

[105] van Driel P.B.A.A., Boonstra M.C., Slooter M.D., Heukers R., Stammes M.A., Snoeks T.J.A., de Bruijn H.S., van Diest P.J., Vahrmeijer A.L., van Bergen en Henegouwen P.M.P. (2016). EGFR targeted nanobody–photosensitizer conjugates for photodynamic therapy in a pre-clinical model of head and neck cancer. J. Control. Release.

[106] Low P.S., Antony A.C. (2004). Folate receptor-targeted drugs for cancer and inflammatory diseases. Adv. Drug Deliv. Rev..

[107] Leamon C., Low P. (1994). Selective Targeting of Malignant Cells with Cytotoxin-Folate Conjugates. J. Drug Target..

[108] Yang M., Deng J., Guo D., Zhang J., Yang L., Wu F. (2019). A folate-conjugated platinum porphyrin complex as a new cancer-targeting photosensitizer for photodynamic therapy. Org. Biomol. Chem..

[109] Tang Y., Li Y., Xu R., Li S., Hu H., Xiao C., Wu H., Zhu L., Ming J., Chu Z. (2018). Self-assembly of folic acid dextran conjugates for cancer chemotherapy. Nanoscale.

[110] Yoo J., Park C., Yi G., Lee D., Koo H. (2019). Active Targeting Strategies Using Biological Ligands for Nanoparticle Drug Delivery Systems. Cancers.

[111] Rogers L., Majer F., Sergeeva N.N., Paszko E., Gilmer J.F., Senge M.O. (2013). Synthesis and biological evaluation of Foscan® bile acid conjugates to target esophageal cancer cells. Bioorg. Med. Chem. Lett..

[112] Morita Y., Leslie M., Kameyama H., Volk D.E., Tanaka T. (2018). Aptamer Therapeutics in Cancer: Current and Future. Cancers.

[113] Farokhzad O.C., Jon S., Khademhosseini A., Tran T.-N.T., LaVan D.A., Langer R. (2004). Nanoparticle-Aptamer Bioconjugates. Cancer Res..

[114] Kim J., Park W., Kim D., Lee E.S., Lee D.H., Jeong S., Park J.M., Na K. (2019). Tumor-Specific Aptamer-Conjugated Polymeric Photosensitizer for Effective Endo-Laparoscopic Photodynamic Therapy. Adv. Funct. Mater..

[115] Yang Y., Zhu W., Cheng L., Cai R., Yi X., He J., Pan X., Yang L., Yang K., Liu Z. (2020). Tumor microenvironment (TME)-activatable circular aptamer-PEG as an effective hierarchical-targeting molecular medicine for photodynamic therapy. Biomaterials.

[116] Smith G. (1985). Filamentous fusion phage: Novel expression vectors that display cloned antigens on the virion surface. Science.

[117] (2018). Nobel Media AB 2020 The Nobel Prize in Chemistry. https://www.nobelprize.org/prizes/chemistry/2018/prize-announcement/.

[118] Haeri A., Zalba S., ten Hagen T.L.M., Dadashzadeh S., Koning G.A. (2016). EGFR targeted thermosensitive liposomes: A novel multifunctional platform for simultaneous tumor targeted and stimulus responsive drug delivery. Colloid. Surf. B.

[119] Chen J., Ouyang J., Chen Q., Deng C., Meng F., Zhang J., Cheng R., Lan Q., Zhong Z. (2017). EGFR and CD44 Dual-Targeted Multifunctional Hyaluronic Acid Nanogels Boost Protein Delivery to Ovarian and Breast Cancers In Vitro and In Vivo. ACS Appl. Mater. Interfaces.

[120] Colzani B., Speranza G., Dorati R., Conti B., Modena T., Bruni G., Zagato E., Vermeulen L., Dakwar G.R., Braeckmans K. (2016). Design of smart GE11-PLGA/PEG-PLGA blend nanoparticulate platforms for parenteral administration of hydrophilic macromolecular drugs: Synthesis, preparation and in vitro/ex vivo characterization. Int. J. Pharm..

[121] Wang A., Cui M., Qu H., Di J., Wang Z., Xing J., Wu F., Wu W., Wang X., Shen L. (2016). Induction of anti-EGFR immune response with mimotopes identified from a phage display peptide library by panitumumab. Oncotarget.

[122] Sun J., Zhang C., Liu G., Liu H., Zhou C., Lu Y., Zhou C., Yuan L., Li X. (2012). A novel mouse CD133 binding-peptide screened by phage display inhibits cancer cell motility in vitro. Clin. Exp. Metastasis.

[123] De Groof T.W.M., Mashayekhi V., Fan T.S., Bergkamp N.D., Sastre Toraño J., van Senten J.R., Heukers R., Smit M.J., Oliveira S. (2019). Nanobody-Targeted Photodynamic Therapy Selectively Kills Viral GPCR-Expressing Glioblastoma Cells. Mol. Pharm..

[124] Gallo E., Kelil A., Bayliss P.E., Jeganathan A., Egorova O., Ploder L., Adams J.J., Giblin P., Sidhu S.S. (2020). *In situ* antibody phage display yields optimal inhibitors of integrin α11/β1. mAbs.

[125] Nikfarjam S., Tohidkia M.R., Mehdipour T., Soleimani R., Rahimi A.A.R., Nouri M. (2019). Successful Application of Whole Cell Panning for Isolation of PhageAntibody Fragments Specific to Differentiated Gastric Cancer Cells. Adv. Pharm. Bull..

[126] Ferreira D., Silva A.P., Nobrega F.L., Martins I.M., Barbosa-Matos C., Granja S., Martins S.F., Baltazar F., Rodrigues L.R. (2019). Rational Identification of a Colorectal Cancer Targeting Peptide through Phage Display. Sci. Rep..

[127] Pasqualini R., Ruoslahti E. (1996). Organ targeting In vivo using phage display peptide libraries. Nature.

[128] Liu X., Peng J., He J., Li Q., Zhou J., Liang X., Tang S. (2018). Selection and identification of novel peptides specifically targeting human cervical cancer. Amino Acids.

[129] Hassan Baig M., Ahmad K., Roy S., Mohammad Ashraf J., Adil M., Haris Siddiqui M., Khan S., Amjad Kamal M., Provazník I., Choi I. (2016). Computer Aided Drug Design: Success and Limitations. Curr. Pharm. Des..

[130] Hidayat A.T., Yusuf M., Bachti H.H., Diantini A., Zainuddin A. (2018). Computational Model of Doxorubicin Conjugate with Docosahexaenoic Acid and Integrin avß3 Ligand for Anticancer. J. Appl. Pharm. Sci..

[131] Hudson R., Boyle R.W. (2004). Strategies for selective delivery of photodynamic sensitisers to biological targets. J. Porphyrins Phthalocyanines.

[132] Muro S. (2012). Challenges in design and characterization of ligand-targeted drug delivery systems. J. Control. Release.

[133] Stallivieri A., Colombeau L., Jetpisbayeva G., Moussaron A., Myrzakhmetov B., Arnoux P., Acherar S., Vanderesse R., Frochot C. (2017). Folic acid conjugates with photosensitizers for cancer targeting in photodynamic therapy: Synthesis and photophysical properties. Bioorg. Med. Chem..

[134] Suvorov N.V., Mironov A.F., Grin M.A. (2017). Folic acid and its derivatives for targeted photodynamic therapy of cancer. Russ. Chem. Bull..

[135] Jenni S., Sour A., Bolze F., Ventura B., Heitz V. (2019). Tumour-targeting photosensitisers for one- and two-photon activated photodynamic therapy. Org. Biomol. Chem..

[136] Liu Q., Wang J., Li S., Li G., Chen Q., Hong Z. (2019). Folate-Targeted Polyethylene Glycol–Modified Photosensitizers for Photodynamic Therapy. J Pharm. Sci..

[137] Hamblin M.R., Newman E.L. (1994). Photosensitizer targeting in photodynamic therapy I. Conjugates of haematoporphyrin with albumin and transferrin. J. Photochem. Photobiol. B Biol..

[138] Kaspler P., Lazic S., Forward S., Arenas Y., Mandel A., Lilge L. (2016). A ruthenium( ii ) based photosensitizer and transferrin complexes enhance photo-physical properties, cell uptake, and photodynamic therapy safety and efficacy. Photochem. Photobiol. Sci..

[139] Jadia R., Kydd J., Rai P. (2018). Remotely Phototriggered, Transferrin-Targeted Polymeric Nanoparticles for the Treatment of Breast Cancer. Photochem. Photobiol..

[140] Dixit S., Novak T., Miller K., Zhu Y., Kenney M.E., Broome A.-M. (2015). Transferrin receptor-targeted theranostic gold nanoparticles for photosensitizer delivery in brain tumors. Nanoscale.

[141] Zhu X., Zhou H., Liu Y., Wen Y., Wei C., Yu Q., Liu J. (2018). Transferrin/aptamer conjugated mesoporous ruthenium nanosystem for redox-controlled and targeted chemo-photodynamic therapy of glioma. Acta Biomater..

[142] Mitsunaga M., Ogawa M., Kosaka N., Rosenblum L.T., Choyke P.L., Kobayashi H. (2011). Cancer cell-selective in vivo near infrared photoimmunotherapy targeting specific membrane molecules. Nat. Med..

[143] Railkar R., Krane L.S., Li Q.Q., Sanford T., Siddiqui M.R., Haines D., Vourganti S., Brancato S.J., Choyke P.L., Kobayashi H. (2017). Epidermal Growth Factor Receptor (EGFR)-targeted Photoimmunotherapy (PIT) for the Treatment of EGFR-expressing Bladder Cancer. Mol. Cancer Ther..

[144] Siddiqui M.R., Railkar R., Sanford T., Crooks D.R., Eckhaus M.A., Haines D., Choyke P.L., Kobayashi H., Agarwal P.K. (2019). Targeting Epidermal Growth Factor Receptor (EGFR) and Human Epidermal Growth Factor Receptor 2 (HER2) Expressing Bladder Cancer Using Combination Photoimmunotherapy (PIT). Sci. Rep..

[145] Nagaya T., Nakamura Y., Okuyama S., Ogata F., Maruoka Y., Choyke P.L., Kobayashi H. (2017). Near-Infrared Photoimmunotherapy Targeting Prostate Cancer with Prostate-Specific Membrane Antigen (PSMA) Antibody. Mol. Cancer Res..

[146] Watanabe R., Hanaoka H., Sato K., Nagaya T., Harada T., Mitsunaga M., Kim I., Paik C.H., Wu A.M., Choyke P.L. (2015). Photoimmunotherapy targeting prostate-specific membrane antigen: Are antibody fragments as effective as antibodies?. J. Nucl. Med..

[147] Nagaya T., Nakamura Y., Okuyama S., Ogata F., Maruoka Y., Choyke P.L., Allen C., Kobayashi H. (2017). Syngeneic Mouse Models of Oral Cancer Are Effectively Targeted by Anti-CD44-Based NIR-PIT. Mol. Cancer Res..

[148] Isobe Y., Sato K., Nishinaga Y., Takahashi K., Taki S., Yasui H., Shimizu M., Endo R., Koike C., Kuramoto N. (2020). Near infrared photoimmunotherapy targeting DLL3 for small cell lung cancer. EBioMedicine.

[149] Jing H., Weidensteiner C., Reichardt W., Gaedicke S., Zhu X., Grosu A.-L., Kobayashi H., Niedermann G. (2016). Imaging and Selective Elimination of Glioblastoma Stem Cells with Theranostic Near-Infrared-Labeled CD133-Specific Antibodies. Theranostics.

[150] Wei W., Jiang D., Ehlerding E.B., Barnhart T.E., Yang Y., Engle J.W., Luo Q., Huang P., Cai W. (2019). CD146-Targeted Multimodal Image-Guided Photoimmunotherapy of Melanoma. Adv. Sci..

[151] Sato K., Ando K., Okuyama S., Moriguchi S., Ogura T., Totoki S., Hanaoka H., Nagaya T., Kokawa R., Takakura H. (2018). Photoinduced Ligand Release from a Silicon Phthalocyanine Dye Conjugated with Monoclonal Antibodies: A Mechanism of Cancer Cell Cytotoxicity after Near-Infrared Photoimmunotherapy. ACS Cent. Sci..

[152] Kobayashi H. (2020). Near infrared photoimmunotherapy: A new type of immune theranostic technology for cancer. Proc. SPIE.

[153] Kobayashi H., Choyke P.L. (2019). Near-Infrared Photoimmunotherapy of Cancer. Acc. Chem. Res..

[154] ClinicalTrials.gov Study of RM-1929 and Photoimmunotherapy in Patients with Recurrent Head and Neck Cancer. https://clinicaltrials.gov/ct2/show/NCT02422979.

[155] ClinicalTrials.gov ASP-1929 Photoimmunotherapy (PIT) Study in Recurrent Head/Neck Cancer for Patients Who Have Failed at Least Two Lines of Therapy. https://clinicaltrials.gov/ct2/show/NCT03769506.

[156] Aung W., Tsuji A.B., Sugyo A., Takashima H., Yasunaga M., Matsumura Y., Higashi T. (2018). Near-infrared photoimmunotherapy of pancreatic cancer using an indocyanine green-labeled anti-tissue factor antibody. World. J. Gastroenterol..

[157] Darwish W.M., Bayoumi N.A., El-Shershaby H.M., Allahloubi N.M. (2020). Targeted photoimmunotherapy based on photosensitizer-antibody conjugates for multiple myeloma treatment. J. Photochem. Photobiol. B Biol..

[158] Heukers R., Mashayekhi V., Ramirez-Escudero M., de Haard H., Verrips T., van Bergen en Henegouwen P., Oliveira S. (2019). VHH-Photosensitizer Conjugates for Targeted Photodynamic Therapy of Met-Overexpressing Tumor Cells. Antibodies.

[159] Heukers R., Fan T.S., de Wit R.H., van Senten J.R., De Groof T.W.M., Bebelman M.P., Lagerweij T., Vieira J., de Munnik S.M., Smits-de Vries L. (2018). The constitutive activity of the virally encoded chemokine receptor US28 accelerates glioblastoma growth. Oncogene.

[160] Yamaguchi H., Pantarat N., Suzuki T., Evdokiou A. (2019). Near-Infrared Photoimmunotherapy Using a Small Protein Mimetic for HER2-Overexpressing Breast Cancer. Int. J. Mol. Sci..

[161] Yu L., Wang Q., Wong R.C.-H., Zhao S., Ng D.K.P., Lo P.-C. (2019). Synthesis and biological evaluation of phthalocyanine-peptide conjugate for EGFR-targeted photodynamic therapy and bioimaging. Dyes Pigments.

[162] Williams T.M., Sibrian-Vazquez M., Vicente M.G.H., Pandey R.K., Kessel D., Dougherty T.J. (2016). Design and Synthesis of Photosensitizer-Peptide Conjugates for PDT. Handbook of Photodynamic Therapy.

[163] Kim J., Chae J., Kim J.S., Goh S.-H., Choi Y. (2016). Photosensitizer-conjugated tryptophan-containing peptide ligands as new dual-targeted theranostics for cancers. Int. J. Pharm..

[164] Kim J., Won Y., Goh S.-H., Choi Y. (2016). A redox-responsive theranostic agent for target-specific fluorescence imaging and photodynamic therapy of EGFR-overexpressing triple-negative breast cancers. J. Mater. Chem. B.

[165] Xu P., Jia Y., Yang Y., Chen J., Hu P., Chen Z., Huang M. (2017). Photodynamic Oncotherapy Mediated by Gonadotropin-Releasing Hormone Receptors. J. Med. Chem..

[166] Pethő L., Murányi J., Pénzes K., Gurbi B., Brauswetter D., Halmos G., Csík G., Mező G. (2019). Suitability of GnRH Receptors for Targeted Photodynamic Therapy in Head and Neck Cancers. Int. J. Mol. Sci..

[167] Zhang C., Gao F., Wu W., Qiu W.-X., Zhang L., Li R., Zhuang Z.-N., Yu W., Cheng H., Zhang X.-Z. (2019). Enzyme-Driven Membrane-Targeted Chimeric Peptide for Enhanced Tumor Photodynamic Immunotherapy. ACS Nano.

[168] Isaac-Lam M., Hammonds D. (2017). Biotinylated Chlorin and Its Zinc and Indium Complexes: Synthesis and In Vitro Biological Evaluation for Photodynamic Therapy. Pharmaceuticals.

[169] Balçik-Erçin P., Çetin M., Göksel M., Durmuş M. (2020). Improved targeting for photodynamic therapy *via* a biotin–phthalocyanine conjugate: Synthesis, photophysical and photochemical measurements, and *in vitro* cytotoxicity assay. New J. Chem..

[170] Li J., Zeng L., Xiong K., Rees T.W., Jin C., Wu W., Chen Y., Ji L., Chao H. (2019). A biotinylated ruthenium( ii ) photosensitizer for tumor-targeted two-photon photodynamic therapy. Chem. Commun..

[171] Li K., Dong W., Liu Q., Lv G., Xie M., Sun X., Qiu L., Lin J. (2019). A biotin receptor-targeted silicon(IV) phthalocyanine for in vivo tumor imaging and photodynamic therapy. J. Photochem. Photobiol. B Biol..

[172] Osati S., Ali H., Guérin B., van Lier J.E. (2017). Steroid-photosensitizer conjugates: Syntheses and applications. J. Porphyrins Phthalocyanines.

[173] El-Akra N., Noirot A., Faye J.-C., Souchard J.-P. (2006). Synthesis of estradiol–pheophorbide a conjugates: Evidence of nuclear targeting, DNA damage and improved photodynamic activity in human breast cancer and vascular endothelial cells. Photochem. Photobiol. Sci..

[174] Zolottsev V.A., Ponomarev G.V., Taratynova M.O., Morozevich G.E., Novikov R.A., Timofeev V.P., Solyev P.N., Zavialova M.G., Zazulina O.V., Tkachev Y.V. (2018). Conjugates of 17-substituted testosterone and epitestosterone with pyropheophorbide a differing in the length of linkers. Steroids.

[175] Tanaka M., Kataoka H., Mabuchi M., Sakuma S., Takahashi S., Tujii R., Akashi H., Ohi H., Yano S., Morita A. (2011). Anticancer Effects of Novel Photodynamic Therapy with Glycoconjugated Chlorin for Gastric and Colon Cancer. Anticancer Res..

[176] Kataoka H., Nishie H., Hayashi N., Tanaka M., Nomoto A., Yano S., Joh T. (2017). New photodynamic therapy with next-generation photosensitizers. Ann. Transl. Med..

[177] Kruspe S., Meyer C., Hahn U. (2014). Chlorin e6 Conjugated Interleukin-6 Receptor Aptamers Selectively Kill Target Cells Upon Irradiation. Mol. Ther. Nucl Acids.

[178] Mallikaratchy P., Tang Z., Tan W. (2008). Cell Specific Aptamer–Photosensitizer Conjugates as a Molecular Tool in Photodynamic Therapy. ChemMedChem.

[179] Hayashi N., Kataoka H., Yano S., Tanaka M., Moriwaki K., Akashi H., Suzuki S., Mori Y., Kubota E., Tanida S. (2015). A Novel Photodynamic Therapy Targeting Cancer Cells and Tumor-Associated Macrophages. Mol. Cancer Ther..

[180] Paszko E., Ehrhardt C., Senge M.O., Kelleher D.P., Reynolds J.V. (2011). Nanodrug applications in photodynamic therapy. Photodiagn. Photodyn. Ther..

[181] Emerich D.F., Thanos C.G. (2006). The pinpoint promise of nanoparticle-based drug delivery and molecular diagnosis. Biomol. Eng..

[182] Schmidt M.M., Wittrup K.D. (2009). A modeling analysis of the effects of molecular size and binding affinity on tumor targeting. Mol. Cancer Ther..

[183] Puri A., Loomis K., Smith B., Lee J.-H., Yavlovich A., Heldman E., Blumenthal R. (2009). Lipid-Based Nanoparticles as Pharmaceutical Drug Carriers: From Concepts to Clinic. Crit. Rev. Ther. Drug Carrier Syst..

[184] Düzgüneş N., Piskorz J., Skupin-Mrugalska P., Goslinski T., Mielcarek J., Konopka K. (2018). Photodynamic therapy of cancer with liposomal photosensitizers. Ther. Deliv..

[185] Schmidt-Erfurth U., Hasan T. (2000). Mechanisms of Action of Photodynamic Therapy with Verteporfin for the Treatment of Age-Related Macular Degeneration. Surv. Ophthalmol..

[186] Bangham A.D., Standish M.M., Watkins J.C. (1965). Diffusion of univalent ions across the lamellae of swollen phospholipids. J. Mol. Biol..

[187] Allen T.M., Cullis P.R. (2013). Liposomal drug delivery systems: From concept to clinical applications. Adv. Drug Deliv. Rev..

[188] Torchilin V.P. (2005). Recent advances with liposomes as pharmaceutical carriers. Nat. Rev. Drug Discov..

[189] Mir Y., Elrington S.A., Hasan T. (2013). A new nanoconstruct for epidermal growth factor receptor-targeted photo-immunotherapy of ovarian cancer. Nanomed. Nanotechn. Biol. Med..

[190] Li Q., Li W., Di H., Luo L., Zhu C., Yang J., Yin X., Yin H., Gao J., Du Y. (2018). A photosensitive liposome with NIR light triggered doxorubicin release as a combined photodynamic-chemo therapy system. J. Control. Release.

[191] Ichikawa K., Hikita T., Maeda N., Yonezawa S., Takeuchi Y., Asai T., Namba Y., Oku N. (2005). Antiangiogenic photodynamic therapy (PDT) by using long-circulating liposomes modified with peptide specific to angiogenic vessels. Biochim. Biophys. Acta BBA Biomembranes.

[192] de Freitas C.F., Montanha M.C., Pellosi D.S., Kimura E., Caetano W., Hioka N. (2019). Biotin-targeted mixed liposomes: A smart strategy for selective release of a photosensitizer agent in cancer cells. Mater. Sci. Eng. C.

[193] Moret F., Scheglmann D., Reddi E. (2013). Folate-targeted PEGylated liposomes improve the selectivity of PDT with meta-tetra(hydroxyphenyl)chlorin (m-THPC). Photochem. Photobiol. Sci..

[194] Feng Q., Wang J., Song H., Zhuo L., Wang G., Liao W., Feng Y., Wei H., Chen Y., Yang Y. (2018). Uptake and light-induced cytotoxicity of hyaluronic acid-grafted liposomes containing porphyrin in tumor cells. J. Drug Deliv. Sci. Technol..

[195] Lovell J.F., Jin C.S., Huynh E., Jin H., Kim C., Rubinstein J.L., Chan W.C.W., Cao W., Wang L.V., Zheng G. (2011). Porphysome nanovesicles generated by porphyrin bilayers for use as multimodal biophotonic contrast agents. Nat. Mater..

[196] Kato T., Jin C.S., Ujiie H., Lee D., Fujino K., Wada H., Hu H., Weersink R.A., Chen J., Kaji M. (2017). Nanoparticle targeted folate receptor 1-enhanced photodynamic therapy for lung cancer. Lung Cancer.

[197] Mehnert W., Mäder K. (2012). Solid lipid nanoparticles: Production, characterization and applications. Adv. Drug Deliv. Rev..

[198] Dai Y., Su J., Wu K., Ma W., Wang B., Li M., Sun P., Shen Q., Wang Q., Fan Q. (2019). Multifunctional Thermosensitive Liposomes Based on Natural Phase-Change Material: Near-Infrared Light-Triggered Drug Release and Multimodal Imaging-Guided Cancer Combination Therapy. ACS Appl. Mater. Interfaces.

[199] Anilkumar T.S., Lu Y.-J., Chen H.-A., Hsu H.-L., Jose G., Chen J.-P. (2019). Dual targeted magnetic photosensitive liposomes for photothermal/photodynamic tumor therapy. J. Magn. Magn. Mater..

[200] Pardeike J., Hommoss A., Müller R.H. (2009). Lipid nanoparticles (SLN, NLC) in cosmetic and pharmaceutical dermal products. Int. J. Pharm..

[201] Ding X., Xu X., Zhao Y., Zhang L., Yu Y., Huang F., Yin D., Huang H. (2017). Tumor targeted nanostructured lipid carrier co-delivering paclitaxel and indocyanine green for laser triggered synergetic therapy of cancer. RSC Adv..

[202] García-Díaz M., Nonell S., Villanueva Á., Stockert J.C., Cañete M., Casadó A., Mora M., Sagristá M.L. (2011). Do folate-receptor targeted liposomal photosensitizers enhance photodynamic therapy selectivity?. Biochim. Biophys. Acta BBA Biomembr..

[203] Liang B.J., Pigula M., Baglo Y., Najafali D., Hasan T., Huang H.-C. (2020). Breaking the selectivity-uptake trade-off of photoimmunoconjugates with nanoliposomal irinotecan for synergistic multi-tier cancer targeting. J. Nanobiotechnol..

[204] Savellano M.D., Owusu-Brackett N., Son J., Ganga T., Leung N.L., Savellano D.H. (2013). Photodynamic Tumor Eradication With a Novel Targetable Photosensitizer: Strong Vascular Effects and Dependence on Treatment Repetition Versus Potentiation. Photochem. Photobiol..

[205] Hu Z., Rao B., Chen S., Duanmu J. (2010). Targeting tissue factor on tumour cells and angiogenic vascular endothelial cells by factor VII-targeted verteporfin photodynamic therapy for breast cancer in vitro and in vivo in mice. BMC Cancer.

[206] Oshiro-Junior J.A., Sato M.R., Boni F.I., Santos K.L.M., de Oliveira K.T., de Freitas L.M., Fontana C.R., Nicholas D., McHale A., Callan J.F. (2020). Phthalocyanine-loaded nanostructured lipid carriers functionalized with folic acid for photodynamic therapy. Mater. Sci. Eng. C.

[207] Li J., Xue Y., Tian J., Liu Z., Zhuang A., Gu P., Zhou H., Zhang W., Fan X. (2020). Fluorinated-functionalized hyaluronic acid nanoparticles for enhanced photodynamic therapy of ocular choroidal melanoma by ameliorating hypoxia. Carbohydr. Polym..

[208] Gaio E., Conte C., Esposito D., Reddi E., Quaglia F., Moret F. (2020). CD44 Targeting Mediated by Polymeric Nanoparticles and Combination of Chlorine TPCS2a-PDT and Docetaxel-Chemotherapy for Efficient Killing of Breast Differentiated and Stem Cancer Cells In Vitro. Cancers.

[209] Gangopadhyay M., Singh T., Behara K.K., Karwa S., Ghosh S.K., Singh N.D.P. (2015). Coumarin-containing-star-shaped 4-arm-polyethylene glycol: Targeted fluorescent organic nanoparticles for dual treatment of photodynamic therapy and chemotherapy. Photochem. Photobiol. Sci..

[210] Rezvantalab S., Drude N.I., Moraveji M.K., Güvener N., Koons E.K., Shi Y., Lammers T., Kiessling F. (2018). PLGA-Based Nanoparticles in Cancer Treatment. Front. Pharmacol..

[211] Zhang Y., Ma N., Luo C., Zhu J., Bao C. (2020). Photosensitizer-loaded cell membrane biomimetic nanoparticles for enhanced tumor synergetic targeted therapy. RSC Adv..

[212] Chen H., Tian J., He W., Guo Z. (2015). H2O2-Activatable and O2-Evolving Nanoparticles for Highly Efficient and Selective Photodynamic Therapy against Hypoxic Tumor Cells. J. Am. Chem. Soc..

[213] Master A., Malamas A., Solanki R., Clausen D.M., Eiseman J.L., Sen Gupta A. (2013). A Cell-Targeted Photodynamic Nanomedicine Strategy for Head and Neck Cancers. Mol. Pharm..

[214] Buwalda S.J., Boere K.W.M., Dijkstra P.J., Feijen J., Vermonden T., Hennink W.E. (2014). Hydrogels in a historical perspective: From simple networks to smart materials. J. Control. Release.

[215] Khurana B., Gierlich P., Meindl A., Gomes-da-Silva L.C., Senge M.O. (2019). Hydrogels: Soft matters in photomedicine. Photochem. Photobiol. Sci..

[216] Belali S., Savoie H., O’Brien J.M., Cafolla A.A., O’Connell B., Karimi A.R., Boyle R.W., Senge M.O. (2018). Synthesis and Characterization of Temperature-Sensitive and Chemically Cross-Linked Poly(*N*-isopropylacrylamide)/Photosensitizer Hydrogels for Applications in Photodynamic Therapy. Biomacromolecules.

[217] Bae B., Na K. (2010). Self-quenching polysaccharide-based nanogels of pullulan/folate-photosensitizer conjugates for photodynamic therapy. Biomaterials.

[218] Abdelghany S.M., Schmid D., Deacon J., Jaworski J., Fay F., McLaughlin K.M., Gormley J.A., Burrows J.F., Longley D.B., Donnelly R.F. (2013). Enhanced Antitumor Activity of the Photosensitizer meso-Tetra(*N*-methyl-4-pyridyl) Porphine Tetra Tosylate through Encapsulation in Antibody-Targeted Chitosan/Alginate Nanoparticles. Biomacromolecules.

[219] Belali S., Karimi A.R., Hadizadeh M. (2018). Cell-specific and pH-sensitive nanostructure hydrogel based on chitosan as a photosensitizer carrier for selective photodynamic therapy. Int. J. Biol. Macromol..

[220] Hah H.J., Kim G., Lee Y.-E.K., Orringer D.A., Sagher O., Philbert M.A., Kopelman R. (2011). Methylene Blue-Conjugated Hydrogel Nanoparticles and Tumor-Cell Targeted Photodynamic Therapy. Macromol. Biosci..

[221] Wang S., Kim G., Lee Y.-E.K., Hah H.J., Ethirajan M., Pandey R.K., Kopelman R. (2012). Multifunctional Biodegradable Polyacrylamide Nanocarriers for Cancer Theranostics—A “See and Treat” Strategy. ACS Nano.

[222] Wang H., Fu Z., Li W., Li Y., Zhao L., Wen L., Zhang J., Wen N. (2019). The synthesis and application of nano doxorubicin-indocyanine green matrix metalloproteinase-responsive hydrogel in chemophototherapy for head and neck squamous cell carcinoma. Int. J. Nanomed..

[223] Martínez-Jothar L., Beztsinna N., van Nostrum C.F., Hennink W.E., Oliveira S. (2019). Selective Cytotoxicity to HER2 Positive Breast Cancer Cells by Saporin-Loaded Nanobody-Targeted Polymeric Nanoparticles in Combination with Photochemical Internalization. Mol. Pharm..

[224] Son J., Yang S.M., Yi G., Roh Y.J., Park H., Park J.M., Choi M.-G., Koo H. (2018). Folate-modified PLGA nanoparticles for tumor-targeted delivery of pheophorbide a in vivo. Biochem. Biophys. Res. Commun..

[225] Clement S., Chen W., Deng W., Goldys E.M. (2018). X-ray radiation-induced and targeted photodynamic therapy with folic acid-conjugated biodegradable nanoconstructs. Int. J. Nanomed..

[226] Wang X., Wang J., Li J., Huang H., Sun X., Lv Y. (2018). Development and evaluation of hyaluronic acid-based polymeric micelles for targeted delivery of photosensitizer for photodynamic therapy in vitro. J. Drug Deliv. Sci. Technol..

[227] Lin X., Yan S.-Z., Qi S.-S., Xu Q., Han S.-S., Guo L.-Y., Zhao N., Chen S.-L., Yu S.-Q. (2017). Transferrin-Modified Nanoparticles for Photodynamic Therapy Enhance the Antitumor Efficacy of Hypocrellin A. Front. Pharmacol..

[228] Gaio E., Conte C., Esposito D., Miotto G., Quaglia F., Moret F., Reddi E. (2018). Co-delivery of Docetaxel and Disulfonate Tetraphenyl Chlorin in One Nanoparticle Produces Strong Synergism between Chemo- and Photodynamic Therapy in Drug-Sensitive and -Resistant Cancer Cells. Mol. Pharm..

[229] Dai J., Li Y., Long Z., Jiang R., Zhuang Z., Wang Z., Zhao Z., Lou X., Xia F., Tang B.Z. (2020). Efficient Near-Infrared Photosensitizer with Aggregation-Induced Emission for Imaging-Guided Photodynamic Therapy in Multiple Xenograft Tumor Models. ACS Nano.

[230] Tian B., Hua S., Liu J. (2020). Cyclodextrin-based delivery systems for chemotherapeutic anticancer drugs: A review. Carbohydr. Polym..

[231] Ben Mihoub A., Larue L., Moussaron A., Youssef Z., Colombeau L., Baros F., Frochot C., Vanderesse R., Acherar S. (2018). Use of Cyclodextrins in Anticancer Photodynamic Therapy Treatment. Molecules.

[232] Laza-Knoerr A.L., Gref R., Couvreur P. (2010). Cyclodextrins for drug delivery. J. Drug Target..

[233] Phua S.Z.F., Xue C., Lim W.Q., Yang G., Chen H., Zhang Y., Wijaya C.F., Luo Z., Zhao Y. (2019). Light-Responsive Prodrug-Based Supramolecular Nanosystems for Site-Specific Combination Therapy of Cancer. Chem. Mater..

[234] Yao X., Li M., Li B., Xue C., Cai K., Zhao Y., Luo Z. (2020). Tumor-targeted upconverting nanoplatform constructed by host-guest interaction for near-infrared-light-actuated synergistic photodynamic-/chemotherapy. Chem. Eng..

[235] Zhang Q., Cai Y., Wang X.-J., Xu J.-L., Ye Z., Wang S., Seeberger P.H., Yin J. (2016). Targeted Photodynamic Killing of Breast Cancer Cells Employing Heptamannosylated β-Cyclodextrin-Mediated Nanoparticle Formation of an Adamantane-Functionalized BODIPY Photosensitizer. ACS Appl. Mater. Interfaces.

[236] Hu Z., Wang C., Zhao F., Xu X., Wang S., Yu L., Zhang D., Huang Y. (2017). Fabrication of a graphene/C60 nanohybrid via γ-cyclodextrin host–guest chemistry for photodynamic and photothermal therapy. Nanoscale.

[237] Tong H., Du J., Li H., Jin Q., Wang Y., Ji J. (2016). Programmed photosensitizer conjugated supramolecular nanocarriers with dual targeting ability for enhanced photodynamic therapy. Chem. Commun..

[238] Zagami R., Rapozzi V., Piperno A., Scala A., Triolo C., Trapani M., Xodo L.E., Monsù Scolaro L., Mazzaglia A. (2019). Folate-Decorated Amphiphilic Cyclodextrins as Cell-Targeted Nanophototherapeutics. Biomacromolecules.

[239] Fu H.-G., Chen Y., Yu Q., Liu Y. (2019). A tumor-targeting Ru/polysaccharide/protein supramolecular assembly with high photodynamic therapy ability. Chem. Commun..

[240] Liang W., Huang Y., Lu D., Ma X., Gong T., Cui X., Yu B., Yang C., Dong C., Shuang S. (2019). β-Cyclodextrin–Hyaluronic Acid Polymer Functionalized Magnetic Graphene Oxide Nanocomposites for Targeted Photo-Chemotherapy of Tumor Cells. Polymers.

[241] Hu X., Gao Z., Tan H., Zhang L. (2019). A pH-Responsive Multifunctional Nanocarrier in the Application of Chemo-Photodynamic Therapy. J. Nanomater..

[242] Theodossiou T.A., Gonçalves A.R., Yannakopoulou K., Skarpen E., Berg K. (2015). Photochemical Internalization of Tamoxifens Transported by a “Trojan-Horse” Nanoconjugate into Breast-Cancer Cell Lines. Angew. Chem. Int. Ed..

[243] Yang Y., Zhang Y.-M., Chen Y., Zhao D., Chen J.-T., Liu Y. (2012). Construction of a Graphene Oxide Based Noncovalent Multiple Nanosupramolecular Assembly as a Scaffold for Drug Delivery. Chem. Eur. J..

[244] Jiang B.-P., Zhou B., Lin Z., Liang H., Shen X.-C. (2019). Recent Advances in Carbon Nanomaterials for Cancer Phototherapy. Chem. Eur. J..

[245] Mohajeri M., Behnam B., Sahebkar A. (2019). Biomedical applications of carbon nanomaterials: Drug and gene delivery potentials. J. Cell. Physiol..

[246] Bartelmess J., Quinn S.J., Giordani S. (2015). Carbon nanomaterials: Multi-functional agents for biomedical fluorescence and Raman imaging. Chem. Soc. Rev..

[247] Hong E.J., Choi D.G., Shim M.S. (2016). Targeted and effective photodynamic therapy for cancer using functionalized nanomaterials. Acta Pharm. Sin. B.

[248] Shi J., Ma R., Wang L., Zhang J., Liu R., Li L., Liu Y., Hou L., Yu X., Gao J. (2013). The application of hyaluronic acid-derivatized carbon nanotubes in hematoporphyrin monomethyl ether-based photodynamic therapy for in vivo and in vitro cancer treatment. Int. J. Nanomed..

[249] Zheng D.-W., Li B., Li C.-X., Fan J.-X., Lei Q., Li C., Xu Z., Zhang X.-Z. (2016). Carbon-Dot-Decorated Carbon Nitride Nanoparticles for Enhanced Photodynamic Therapy against Hypoxic Tumor via Water Splitting. ACS Nano.

[250] Shi J., Wang B., Wang L., Lu T., Fu Y., Zhang H., Zhang Z. (2016). Fullerene (C60)-based tumor-targeting nanoparticles with “off-on” state for enhanced treatment of cancer. J. Control. Release.

[251] Hu Y., Wang R., Zhou Y., Yu N., Chen Z., Gao D., Shi X., Shen M. (2018). Targeted dual-mode imaging and phototherapy of tumors using ICG-loaded multifunctional MWCNTs as a versatile platform. J. Mater. Chem. B.

[252] Li N., Li T., Hu C., Lei X., Zuo Y., Han H. (2016). Targeted Near-Infrared Fluorescent Turn-on Nanoprobe for Activatable Imaging and Effective Phototherapy of Cancer Cells. ACS Appl. Mater. Interfaces.

[253] Mei C., Wang N., Zhu X., Wong K.-H., Chen T. (2018). Photothermal-Controlled Nanotubes with Surface Charge Flipping Ability for Precise Synergistic Therapy of Triple-Negative Breast Cancer. Adv. Funct. Mater..

[254] Ogbodu R.O., Ndhundhuma I., Karsten A., Nyokong T. (2015). Photodynamic therapy effect of zinc monoamino phthalocyanine–folic acid conjugate adsorbed on single walled carbon nanotubes on melanoma cells. Spectrochim. Acta A.

[255] Wang G., Zhang F., Tian R., Zhang L., Fu G., Yang L., Zhu L. (2016). Nanotubes-Embedded Indocyanine Green–Hyaluronic Acid Nanoparticles for Photoacoustic-Imaging-Guided Phototherapy. ACS Appl. Mater. Interfaces.

[256] Chavva S.R., Pramanik A., Nellore B.P.V., Sinha S.S., Yust B., Kanchanapally R., Fan Z., Crouch R.A., Singh A.K., Neyland B. (2014). Theranostic Graphene Oxide for Prostate Cancer Detection and Treatment. Part. Part. Syst. Charact..

[257] Cho Y., Kim H., Choi Y. (2013). A graphene oxide–photosensitizer complex as an enzyme-activatable theranostic agent. Chem. Commun..

[258] Huang P., Wang S., Wang X., Shen G., Lin J., Wang Z., Guo S., Cui D., Yang M., Chen X. (2015). Surface Functionalization of Chemically Reduced Graphene Oxide for Targeted Photodynamic Therapy. J. Biomed. Nanotechnol..

[259] Huang P., Xu C., Lin J., Wang C., Wang X., Zhang C., Zhou X., Guo S., Cui D. (2011). Folic Acid-conjugated Graphene Oxide loaded with Photosensitizers for Targeting Photodynamic Therapy. Theranostics.

[260] Li F., Park S.-J., Ling D., Park W., Han J.Y., Na K., Char K. (2013). Hyaluronic acid-conjugated graphene oxide/photosensitizer nanohybrids for cancer targeted photodynamic therapy. J. Mater. Chem. B.

[261] Liang J., Chen B., Hu J., Huang Q., Zhang D., Wan J., Hu Z., Wang B. (2019). pH and Thermal Dual-Responsive Graphene Oxide Nanocomplexes for Targeted Drug Delivery and Photothermal-Chemo/Photodynamic Synergetic Therapy. ACS Appl. Bio Mater..

[262] Luan X., Guan Y.-Y., Liu H.-J., Lu Q., Zhao M., Sun D., Lovell J.F., Sun P., Chen H.-Z., Fang C. (2018). A Tumor Vascular-Targeted Interlocking Trimodal Nanosystem That Induces and Exploits Hypoxia. Adv. Sci..

[263] Qin X., Wang Z., Guo C., Jin Y. (2020). Multi-responsive drug delivery nanoplatform for tumor-targeted synergistic photothermal/dynamic therapy and chemotherapy. New J. Chem..

[264] Tian J., Ding L., Wang Q., Hu Y., Jia L., Yu J.-S., Ju H. (2015). Folate Receptor-Targeted and Cathepsin B-Activatable Nanoprobe for In Situ Therapeutic Monitoring of Photosensitive Cell Death. Anal. Chem..

[265] Wei Y., Zhou F., Zhang D., Chen Q., Xing D. (2016). A graphene oxide based smart drug delivery system for tumor mitochondria-targeting photodynamic therapy. Nanoscale.

[266] Yang C., Zhang H., Wang Z., Wu X., Jin Y. (2019). Mitochondria-targeted tri-triphenylphosphonium substituted meso-tetra(4-carboxyphenyl)porphyrin(TCPP) by conjugation with folic acid and graphene oxide for improved photodynamic therapy. J. Porphyrins Phthalocyanines.

[267] Yu X., Gao D., Gao L., Lai J., Zhang C., Zhao Y., Zhong L., Jia B., Wang F., Chen X. (2017). Inhibiting Metastasis and Preventing Tumor Relapse by Triggering Host Immunity with Tumor-Targeted Photodynamic Therapy Using Photosensitizer-Loaded Functional Nanographenes. ACS Nano.

[268] Fan J., Fang G., Zeng F., Wang X., Wu S. (2013). Water-Dispersible Fullerene Aggregates as a Targeted Anticancer Prodrug with both Chemo- and Photodynamic Therapeutic Actions. Small.

[269] Hu Z., Zhang C., Huang Y., Sun S., Guan W., Yao Y. (2012). Photodynamic anticancer activities of water-soluble C60 derivatives and their biological consequences in a HeLa cell line. Chem. Biol. Interact..

[270] Kim S., Park J., Youn Y.S., Oh K.T., Bae J.H., Lee E.S. (2015). Hoechst 33258–conjugated hyaluronated fullerene for efficient photodynamic tumor therapy and necrotic tumor targeting. J. Bioact. Compat. Polym..

[271] Liu J., Tabata Y. (2010). Photodynamic therapy of fullerene modified with pullulan on hepatoma cells. J. Drug Target..

[272] Liu J., Tabata Y. (2011). Effect of Modification Manner on the Photodynamic Antitumor Activity of C60 Modified with Pullulan. J. Biomater. Sci. Polym. Ed..

[273] Liu Q., Xu L., Zhang X., Li N., Zheng J., Guan M., Fang X., Wang C., Shu C. (2013). Enhanced Photodynamic Efficiency of an Aptamer-Guided Fullerene Photosensitizer toward Tumor Cells. Chem. Asian J..

[274] Serda M., Ware M.J., Newton J.M., Sachdeva S., Krzykawska-Serda M., Nguyen L., Law J., Anderson A.O., Curley S.A., Wilson L.J. (2018). Development of photoactive Sweet-C60 for pancreatic cancer stellate cell therapy. Nanomedicine.

[275] Shi J., Wang Z., Wang L., Wang H., Li L., Yu X., Zhang J., Ma R., Zhang Z. (2013). Photodynamic therapy of a 2-methoxyestradiol tumor-targeting drug delivery system mediated by Asn-Gly-Arg in breast cancer. Int. J. Nanomed..

[276] Zhang H., Hou L., Jiao X., Ji Y., Zhu X., Zhang Z. (2015). Transferrin-mediated fullerenes nanoparticles as Fe2+-dependent drug vehicles for synergistic anti-tumor efficacy. Biomaterials.

[277] Nicholas D., Fowley C., McHale A.P., Kamila S., Sheng J., Atchison J., Callan J.F. (2015). A folic acid labelled carbon quantum dot-protoporphryin IX conjugate for use in folate receptor targeted two-photon excited photodynamic therapy. Proc. SPIE.

[278] Yao H., Zhao W., Zhang S., Guo X., Li Y., Du B. (2018). Dual-functional carbon dot-labeled heavy-chain ferritin for self-targeting bio-imaging and chemo-photodynamic therapy. J. Mater. Chem. B.

[279] Bhattacharyya S., Kudgus R.A., Bhattacharya R., Mukherjee P. (2011). Inorganic Nanoparticles in Cancer Therapy. Pharm. Res..

[280] Bhattacharya R., Mukherjee P. (2008). Biological properties of “naked” metal nanoparticles. Adv. Drug Deliv. Rev..

[281] Makovec T. (2019). Cisplatin and Beyond: Molecular Mechanisms of Action and Drug Resistance Development in Cancer Chemotherapy. Radiol. Oncol..

[282] Martinelli C., Pucci C., Ciofani G. (2019). Nanostructured carriers as innovative tools for cancer diagnosis and therapy. APL Bioeng..

[283] Cheng S.-H., Lo L.-W. (2011). Inorganic Nanoparticles for Enhanced Photodynamic Cancer Therapy. Curr. Drug Discov. Technol..

[284] Erathodiyil N., Ying J.Y. (2011). Functionalization of Inorganic Nanoparticles for Bioimaging Applications. Acc. Chem. Res..

[285] Wang J., Xu D., Deng T., Li Y., Xue L., Yan T., Huang D., Deng D. (2018). Self-Decomposable Mesoporous Doxorubicin@Silica Nanocomposites for Nuclear Targeted Chemo-Photodynamic Combination Therapy. ACS Appl. Nano Mater..

[286] Xu W., Qian J., Hou G., Wang Y., Wang J., Sun T., Ji L., Suo A., Yao Y. (2019). A dual-targeted hyaluronic acid-gold nanorod platform with triple-stimuli responsiveness for photodynamic/photothermal therapy of breast cancer. Acta Biomater..

[287] Wang P., Shi Y., Zhang S., Huang X., Zhang J., Zhang Y., Si W., Dong X. (2019). Hydrogen Peroxide Responsive Iron–Based Nanoplatform for Multimodal Imaging–Guided Cancer Therapy. Small.

[288] Wu M.-X., Yang Y.-W. (2017). Metal–Organic Framework (MOF)-Based Drug/Cargo Delivery and Cancer Therapy. Adv. Mater..

[289] Lan G., Ni K., Lin W. (2019). Nanoscale metal–organic frameworks for phototherapy of cancer. Coord. Chem. Rev..

[290] Chen Z.-X., Liu M.-D., Zhang M.-K., Wang S.-B., Xu L., Li C.-X., Gao F., Xie B.-R., Zhong Z.-L., Zhang X.-Z. (2018). Interfering with Lactate-Fueled Respiration for Enhanced Photodynamic Tumor Therapy by a Porphyrinic MOF Nanoplatform. Adv. Funct. Mater..

[291] Dam D.H.M., Zhao L., Jelsma S.A., Zhao Y., Paller A.S. (2019). Folic acid functionalized hollow nanoparticles for selective photodynamic therapy of cutaneous squamous cell carcinoma. Mater. Chem. Front..

[292] Bouffard E., Mauriello Jimenez C., El Cheikh K., Maynadier M., Basile I., Raehm L., Nguyen C., Gary-Bobo M., Garcia M., Durand J.-O. (2019). Efficient Photodynamic Therapy of Prostate Cancer Cells through an Improved Targeting of the Cation-Independent Mannose 6-Phosphate Receptor. Int. J. Mol. Sci..

[293] Wang D., Li X., Li X., Kang A., Sun L., Sun M., Yang F., Xu C. (2019). Magnetic and pH dual-responsive nanoparticles for synergistic drug-resistant breast cancer chemo/photodynamic therapy. Int. J. Nanomed..

[294] Park S., Park H., Jeong S., Yi B.G., Park K., Key J. (2019). Hyaluronic Acid-Conjugated Mesoporous Silica Nanoparticles Loaded with Dual Anticancer Agents for Chemophotodynamic Cancer Therapy. J. Nanomater..

[295] Chen C., Zhou L., Geng J., Ren J., Qu X. (2013). Photosensitizer-Incorporated Quadruplex DNA-Gated Nanovechicles for Light-Triggered, Targeted Dual Drug Delivery to Cancer Cells. Small.

[296] Selvestrel F., Moret F., Segat D., Woodhams J.H., Fracasso G., Echevarria I.M.R., Baù L., Rastrelli F., Compagnin C., Reddi E. (2013). Targeted delivery of photosensitizers: Efficacy and selectivity issues revealed by multifunctional ORMOSIL nanovectors in cellular systems. Nanoscale.

[297] Ma X., Qu Q., Zhao Y. (2015). Targeted Delivery of 5-Aminolevulinic Acid by Multifunctional Hollow Mesoporous Silica Nanoparticles for Photodynamic Skin Cancer Therapy. ACS Appl. Mater. Interfaces.

[298] Benachour H., Sève A., Bastogne T., Frochot C., Vanderesse R., Jasniewski J., Miladi I., Billotey C., Tillement O., Lux F. (2012). Multifunctional Peptide-Conjugated Hybrid Silica Nanoparticles for Photodynamic Therapy and MRI. Theranostics.

[299] Gary-Bobo M., Brevet D., Benkirane-Jessel N., Raehm L., Maillard P., Garcia M., Durand J.-O. (2012). Hyaluronic acid-functionalized mesoporous silica nanoparticles for efficient photodynamic therapy of cancer cells. Photodiagn. Photodyn. Ther..

[300] Gary-Bobo M., Mir Y., Rouxel C., Brevet D., Hocine O., Maynadier M., Gallud A., Da Silva A., Mongin O., Blanchard-Desce M. (2012). Multifunctionalized mesoporous silica nanoparticles for the in vitro treatment of retinoblastoma: Drug delivery, one and two-photon photodynamic therapy. Int. J. Pharm..

[301] Cheng S.-H., Lee C.-H., Chen M.-C., Souris J.S., Tseng F.-G., Yang C.-S., Mou C.-Y., Chen C.-T., Lo L.-W. (2010). Tri-functionalization of mesoporous silica nanoparticles for comprehensive cancer theranostics—the trio of imaging, targeting and therapy. J. Mater. Chem..

[302] Gary-Bobo M., Hocine O., Brevet D., Maynadier M., Raehm L., Richeter S., Charasson V., Loock B., Morère A., Maillard P. (2012). Cancer therapy improvement with mesoporous silica nanoparticles combining targeting, drug delivery and PDT. Int. J. Pharm..

[303] Chabloz N.G., Perry H.L., Yoon I.-C., Coulson A.J., White A.J.P., Stasiuk G.J., Botnar R.M., Wilton-Ely J.D.E.T. (2020). Combined Magnetic Resonance Imaging and Photodynamic Therapy Using Polyfunctionalised Nanoparticles Bearing Robust Gadolinium Surface Units. Chem. Eur. J..

[304] Paris J.L., Villaverde G., Gómez-Graña S., Vallet-Regí M. (2020). Nanoparticles for multimodal antivascular therapeutics: Dual drug release, photothermal and photodynamic therapy. Acta Biomater..

[305] Li N., Xiang M.-H., Liu J.-W., Tang H., Jiang J.-H. (2018). DNA Polymer Nanoparticles Programmed via Supersandwich Hybridization for Imaging and Therapy of Cancer Cells. Anal. Chem..

[306] Xia F., Hou W., Liu Y., Wang W., Han Y., Yang M., Zhi X., Li C., Qi D., Li T. (2018). Cytokine induced killer cells-assisted delivery of chlorin e6 mediated self-assembled gold nanoclusters to tumors for imaging and immuno-photodynamic therapy. Biomaterials.

[307] Mangadlao J.D., Wang X., McCleese C., Escamilla M., Ramamurthy G., Wang Z., Govande M., Basilion J.P., Burda C. (2018). Prostate-Specific Membrane Antigen Targeted Gold Nanoparticles for Theranostics of Prostate Cancer. ACS Nano.

[308] Bhattacharjee T.T., Castilho M.L., de Oliveira I.R., Jesus V.P.S., Hewitt K.C., Raniero L. (2018). FTIR study of secondary structure changes in Epidermal Growth Factor by gold nanoparticle conjugation. Biochim. Biophys. Acta BBA Gen. Subj..

[309] García Calavia P., Chambrier I., Cook M.J., Haines A.H., Field R.A., Russell D.A. (2018). Targeted photodynamic therapy of breast cancer cells using lactose-phthalocyanine functionalized gold nanoparticles. J. Colloid Interface Sci..

[310] Li H., Wang P., Deng Y., Zeng M., Tang Y., Zhu W.-H., Cheng Y. (2017). Combination of active targeting, enzyme-triggered release and fluorescent dye into gold nanoclusters for endomicroscopy-guided photothermal/photodynamic therapy to pancreatic ductal adenocarcinoma. Biomaterials.

[311] Penon O., Marín M.J., Russell D.A., Pérez-García L. (2017). Water soluble, multifunctional antibody-porphyrin gold nanoparticles for targeted photodynamic therapy. J. Colloid Interface Sci..

[312] Wu J., Han H., Jin Q., Li Z., Li H., Ji J. (2017). Design and Proof of Programmed 5-Aminolevulinic Acid Prodrug Nanocarriers for Targeted Photodynamic Cancer Therapy. ACS Appl. Mater. Interfaces.

[313] Zhao L., Kim T.-H., Kim H.-W., Ahn J.-C., Kim S.Y. (2016). Enhanced cellular uptake and phototoxicity of Verteporfin-conjugated gold nanoparticles as theranostic nanocarriers for targeted photodynamic therapy and imaging of cancers. Mater. Sci. Eng. C.

[314] Dixit S., Miller K., Zhu Y., McKinnon E., Novak T., Kenney M.E., Broome A.-M. (2015). Dual Receptor-Targeted Theranostic Nanoparticles for Localized Delivery and Activation of Photodynamic Therapy Drug in Glioblastomas. Mol. Pharm..

[315] Nair L.V., Nazeer S.S., Jayasree R.S., Ajayaghosh A. (2015). Fluorescence Imaging Assisted Photodynamic Therapy Using Photosensitizer-Linked Gold Quantum Clusters. ACS Nano.

[316] Obaid G., Chambrier I., Cook M.J., Russell D.A. (2012). Targeting the Oncofetal Thomsen–Friedenreich Disaccharide Using Jacalin-PEG Phthalocyanine Gold Nanoparticles for Photodynamic Cancer Therapy. Angew. Chem. Int. Ed..

[317] Sun X., Liu B., Chen X., Lin H., Peng Y., Li Y., Zheng H., Xu Y., Ou X., Yan S. (2019). Aptamer-assisted superparamagnetic iron oxide nanoparticles as multifunctional drug delivery platform for chemo-photodynamic combination therapy. J. Mater. Sci. Mater. Med..

[318] Dehvari K., Lin P.-T., Chang J.-Y. (2018). Fluorescence-guided magnetic nanocarriers for enhanced tumor targeting photodynamic therapy. J. Mater. Chem. B.

[319] Shao C., Shang K., Xu H., Zhang Y., Pei Z., Pei Y. (2018). Facile fabrication of hypericin-entrapped glyconanoparticles for targeted photodynamic therapy. Int. J. Nanomed..

[320] Lee J., Kim K.S., Na K. (2016). Caffeic acid-coated multifunctional magnetic nanoparticles for the treatment and bimodal imaging of tumours. J. Photochem. Photobiol. B Biol..

[321] Wang D., Fei B., Halig L.V., Qin X., Hu Z., Xu H., Wang Y.A., Chen Z., Kim S., Shin D.M. (2014). Targeted Iron-Oxide Nanoparticle for Photodynamic Therapy and Imaging of Head and Neck Cancer. ACS Nano.

[322] Cai Z., Xin F., Wei Z., Wu M., Lin X., Du X., Chen G., Zhang D., Zhang Z., Liu X. (2020). Photodynamic Therapy Combined with Antihypoxic Signaling and CpG Adjuvant as an In Situ Tumor Vaccine Based on Metal–Organic Framework Nanoparticles to Boost Cancer Immunotherapy. Adv. Healthc. Mater..

[323] Chen P., Huang Y.-F., Xu G.-Y., Xue J.-P., Chen J.-J. (2019). Functionalized Eu(III)-based nanoscale metal-organic framework for enhanced targeted anticancer therapy. J. Porphyrins Phthalocyanines.

[324] Cheng H., Zhu J.-Y., Li S.-Y., Zeng J.-Y., Lei Q., Chen K.-W., Zhang C., Zhang X.-Z. (2016). An O2 Self-Sufficient Biomimetic Nanoplatform for Highly Specific and Efficient Photodynamic Therapy. Adv. Funct. Mater..

[325] Gong M., Yang J., Zhuang Q., Li Y., Gu J. (2020). Mitochondria-Targeted Nanoscale MOFs for Improved Photodynamic Therapy. ChemNanoMat.

[326] Jia J., Zhang Y., Zheng M., Shan C., Yan H., Wu W., Gao X., Cheng B., Liu W., Tang Y. (2018). Functionalized Eu(III)-Based Nanoscale Metal–Organic Framework To Achieve Near-IR-Triggered and -Targeted Two-Photon Absorption Photodynamic Therapy. Inorg. Chem..

[327] Zhu W., Liu Y., Yang Z., Zhang L., Xiao L., Liu P., Wang J., Yi C., Xu Z., Ren J. (2018). Albumin/sulfonamide stabilized iron porphyrin metal organic framework nanocomposites: Targeting tumor hypoxia by carbonic anhydrase IX inhibition and T1–T2 dual mode MRI guided photodynamic/photothermal therapy. J. Mater. Chem. B.

[328] Zhu W., Zhang L., Yang Z., Liu P., Wang J., Cao J., Shen A., Xu Z., Wang J. (2019). An efficient tumor-inducible nanotheranostics for magnetic resonance imaging and enhanced photodynamic therapy. Chem. Eng. J..

[329] Zhang Y., Wang Q., Chen G., Shi P. (2019). DNA-Functionalized Metal–Organic Framework: Cell Imaging, Targeting Drug Delivery and Photodynamic Therapy. Inorg. Chem..

[330] Mao C., Qu P., Miley M.J., Zhao Y., Li Z., Ming X. (2018). P-glycoprotein targeted photodynamic therapy of chemoresistant tumors using recombinant Fab fragment conjugates. Biomater. Sci..

